# Microexon gene transcriptional profiles and evolution provide insights into blood processing by the *Schistosoma japonicum* esophagus

**DOI:** 10.1371/journal.pntd.0006235

**Published:** 2018-02-12

**Authors:** Xiao-Hong Li, Ricardo DeMarco, Leandro X. Neves, Sally R. James, Katherine Newling, Peter D. Ashton, Jian-Ping Cao, R. Alan Wilson, William Castro-Borges

**Affiliations:** 1 National Institute of Parasitic Diseases, Chinese Center for Disease Control and Prevention, Shanghai, People’s Republic of China; 2 Instituto de Física de São Carlos, Universidade de São Paulo, Sao Carlos, Brasil; 3 Departamento de Ciências Biológicas, Universidade Federal de Ouro Preto, Campus Morro do Cruzeiro, Ouro Preto, Minas Gerais, Brasil; 4 Genomics and Bioinformatics Laboratory, Department of Biology, University of York, York, United Kingdom; 5 Centre for Immunology and Infection, Department of Biology, University of York, York, United Kingdom; Queen's University Belfast, UNITED KINGDOM

## Abstract

**Background:**

Adult schistosomes have a well-developed alimentary tract comprising an oral sucker around the mouth, a short esophagus and a blind ending gut. The esophagus is not simply a muscular tube for conducting blood from the mouth to gut but is divided into compartments, surrounded by anterior and posterior glands, where processing of ingested blood is initiated. Self-cure of rhesus macaques from a *Schistosoma japonicum* infection appears to operate by blocking the secretory functions of these glands so that the worms cease feeding and slowly starve to death. Here we use subtractive RNASeq to characterise the genes encoding the principal secretory products of *S*. *japonicum* esophageal glands, preparatory to evaluating their relevance as targets of the self-cure process.

**Methodology/Principal findings:**

The heads and a small portion of the rear end of male and female *S*. *japonicum* worms were separately enriched by microdissection, for mRNA isolation and library construction. The sequence reads were then assembled *de novo* using Trinity and those genes enriched more than eightfold in the head preparation were subjected to detailed bioinformatics analysis. Of the 62 genes selected from the male heads, more than one third comprised MEGs encoding secreted or membrane-anchored proteins. Database searching using conserved motifs revealed that the MEG-4 and MEG-8/9 families had counterparts in the bird schistosome *Trichobilharzia regenti*, indicating an ancient association with blood processing. A second group of MEGs, including a MEG-26 family, encoded short peptides with amphipathic properties that most likely interact with ingested host cell membranes to destabilise them. A number of lysosomal hydrolases, two protease inhibitors, a secreted VAL and a putative natterin complete the line-up. There was surprisingly little difference between expression patterns in males and females despite the latter processing much more blood.

**Significance/Conclusions:**

The mixture of approximately 40 proteins specifically secreted by the esophageal glands is responsible for initiating blood processing in the adult worm esophagus. They comprise the potential targets for the self-cure process in the rhesus macaque, and thus represent a completely new cohort of secreted proteins that can be investigated as vaccine candidates.

## Author summary

Schistosomes are parasitic flatworms inhabiting the human bloodstream, surrounded by and feeding on humoral and cellular components of the immune system. They are normally long-lived but the rhesus macaque is able to mount a self-cure response directed against the esophageal secretions of the adult *Schistosoma japonicum* so that they stop feeding and slowly starve to death. The worm esophagus is a short tube connecting mouth to gut surrounded by anterior and posterior glands and our aim in this study was to identify the genes encoding the gland secretions. For this purpose we isolated the messenger RNA from both male and female worm heads and tails and obtained many millions of sequences. These were assembled into gene coding sequences using bioinformatics and then genes differentially expressed in the head region were identified by a subtraction process. We then focused on those genes encoding proteins with a leader sequence indicating their secretory status. The result is an inventory of approximately 40 genes; some encode protein binding motifs while others encode a short helix with a hydrophobic face, which may interact with host cell membranes. Genes encoding enzymes, protease inhibitors and a venom-like protein were also found. These proteins are being evaluated for their interactions with the antibodies generated by macaques during the self-cure process.

## Introduction

Adult schistosome worms dwell in the hepatic portal system (*Schistosoma mansoni* and *S*. *japonicum*) or the venous plexuses around the bladder (*S*. *haematobium*) vigorously ingesting blood. Unusual among parasitic helminths, they take up nutrients in two distinct ways: active feeding via the alimentary tract and absorption across the body surface. While both routes contribute to nutrient uptake in the two sexes, there is a remarkable dichotomy between male and female schistosomes in the respective importance of the gut and tegument in nutrient acquisition [1, 2]. The blind-ending gut occupies a much greater proportion of body cross section and the alimentary route plays a more important role in the female than in the male [2]. The alimentary tract comprises an oral sucker around the mouth, a short esophagus and an extended gut caecum that runs to the extreme posterior [2]. Much information on nutrient acquisition in the gut caecum has been obtained from recent studies using proteomic analysis of vomitus (reviewed in [2]), in vitro feeding experiments [3], and laser capture microdissection [4]. In contrast, the role of the esophagus has received scant attention since the first ultrastructural descriptions several decades ago [5, 6]. Our recent studies have shown that, instead of being just a food conduit, the esophagus of schistosomes is the initiator of blood processing [7]. Erythrocytes are rapidly lysed upon entry to the posterior esophageal lumen [7, 8] while host leukocytes are tethered there as a central plug around which incoming blood flows [7]. These tethered leukocytes are structurally damaged after capture, indicating the existence of parasite-derived cytolytic mechanisms. Additionally, intact platelets have been observed in the lumen [9] but ingested blood does not clot, implying there is an anticoagulant effect.

The schistosome esophagus is divided into anterior and posterior compartments, each surrounded by an associated mass of cell bodies and lined by a syncytial layer of cytoplasm continuous with the surface tegument. The posterior mass was designated as a gland decades ago and we have shown, in *S*. *japonicum*, that the anterior cell mass is also a distinct secretory organ [10]. Both cell masses synthesise proteins for secretion into the lumen. Three microexon genes (MEGs), namely MEG-4.1 [11], MEG-4.2 and MEG-14 [7], and one venom-allergen-like (VAL) gene, VAL-7 [12] were the first to be localized to the posterior esophageal gland of *S*. *mansoni* by whole mount in-situ hybridisation (WISH). Subsequently, we have used subtractive RNA-Seq on head and tail preparations of *S*. *mansoni* males to pinpoint genes predominantly or exclusively expressed in the heads [13]. WISH revealed that 12 selected genes were uniquely expressed in either the anterior or posterior esophageal glands. The largest group encoding potentially secreted esophageal proteins comprised 27 MEGs, while a smaller group comprised nine hydrolytic enzymes of lysosomal origin.

Much less is known about the composition of esophageal secretions in *S*. *japonicum* which, with its wide host range, is considered to be more basal in the phylogenetic tree [14]. It is also more pathogenic than *S*. *mansoni* due to the much greater egg output per female (2000/day versus 300/day, respectively; [2]). Only seven proteins have been localized to the posterior gland by immunocytochemistry (six MEGs and VAL-7) [7, 15]. Discovering *S*. *japonicum* orthologues for the *S*. *mansoni* esophageal MEGs is problematic due to the fragmented state of the *S*. *japonicum* genome assembly. The situation is compounded by the large orthologous variation occurring between genes encoding secreted MEGs (and VALs), which have accumulated the largest number of non-synonymous nucleotide substitutions [16]. Indeed, this led us to suggest that MEGs and VALs are among the most rapidly evolving gene families in schistosomes, potentially as a result of selection pressure exerted by the immune system on their protein products [16]. In the current study, we have used the subtractive RNA-Seq approach to characterize the gene expression pattern in the esophagus of *S*. *japonicum*. We sought differences between males and females that might provide a better understanding of blood processing in females, given the much greater amounts of blood they ingest. We have also explored the evolution of MEGs, the major group of gene expressed in the esophagus, using bioinformatic analysis.

## Materials and methods

### Ethics statement

Animal care and all animal procedures were carried out in compliance with the Guidelines for the Care and Use of Laboratory Animals produced by the Shanghai Veterinary Research Institute. The study was approved by the Ethics Committee of the Institute of Parasitic Diseases, Chinese Center for Disease Control and Prevention (ID SYXK 2016–00196).

### Biological material

Cercariae of *S*. *japonicum* were shed from naturally infected *Oncomelania hupensis* snails collected from fields in Anhui Province, P.R. China and white New Zealand rabbits were infected percutaneously with 1,000 larvae. Adult worms were obtained by portal perfusion of animals at 5–6 weeks, using RPMI-1640 medium buffered with 10mM HEPES, pH 7.4 (Sigma-Aldrich, St Louis, MO, USA). After extensive washing in the same medium and removal of tissue debris and any damaged individuals under a dissecting microscope, parasites were “fixed” by immersion in RNAlater (Invitrogen, Paisley, UK) and stored in this solution at 4C. The head regions of approximately 400 male worms were individually microdissected, as previously described [13], along with 200 tails, defined as the posterior third of the male body to exclude the testes, to provide the same amount of biological material (S1 Fig). Approximately 100 of the much smaller female heads, plus short sections of tails containing vitellaria were also prepared, but processed and sequenced by more sensitive techniques. Before RNA extraction, all sample pools were disrupted on ice using a tissue grinder until they appeared completely homogeneous.

### Total RNA isolation

Total RNA was extracted from samples using an RNeasy Micro kit (Qiagen, Manchester, UK). Briefly, the homogenized lysate was centrifuged for 3 min at full speed to pellet the debris. The supernatant was transferred to a clean tube and mixed with 1 volume of 70% ethanol. The mixture was then transferred to an RNeasy MinElute spin column and centrifuged for 15s at ≥8000xg. After washing and DNA digestion with DNase I, total RNA was eluted with 10μl RNase free water. RNA quality and quantity were assessed using an Agilent Bioanalyzer (Agilent Technologies, Cheadle, UK).

### mRNA sequencing of male heads and tails

Total RNA was used to prepare mRNA sequencing libraries using the NEBNext RNA Ultra Library prep kit in conjunction with the NEBNext Poly(A) mRNA Magnetic Isolation Module (New England BioLabs Inc.), according to the manufacturer’s instructions, with the modified protocol for longer read lengths (for sequencing insert sizes of 300–600 bp). Briefly, mRNA was purified from a 200 ng sample of good quality total RNA using two rounds of sample binding to oligo d(T)-coupled paramagnetic beads and washing. Purified mRNA was eluted from the beads into a first strand synthesis reaction buffer plus random primer mix, incubating at 94°C to fragment RNA. After addition of RNase inhibitors and ProtoScript II Reverse Transcriptase, first strand cDNA synthesis was performed by incubating at 10 minutes at 25°C, 50 minutes at 42°C then 15 minutes at 70°C. Second strand synthesis and sample clean up were performed according to the manufacturer’s guidelines, as were subsequent end preparation and adapter ligation steps. Libraries were amplified and barcoding indices added in a 13 cycle PCR reaction. Following a final clean up step, the yield and size distribution of each amplified cDNA library were assessed using the Agilent High Sensitivity DNA kit with the Agilent 2100 Bioanalyzer and quantified using the Qubit with a HS dsDNA kit (Thermo Fisher Scientific, Loughborough, UK). Libraries were then pooled at equimolar concentrations, and sequenced on a MiSeq (Illumina, San Diego, CA) using a MiSeq v2 500 cycle reagent kit, running 2 x 250 cycles of sequencing.

### mRNA sequencing of female heads and tails

cDNA synthesis and amplification from polyA transcripts were performed on 10 ng total RNA using the SMART-Seq v4 Ultra Low Input RNA Kit for Sequencing (Takara Bio, Clontech Laboratories), according to the manufacturer’s instructions and using 11 cycles of PCR amplification. Successful cDNA synthesis was confirmed by running samples on an Agilent 2100 Bioanalyzer using the Agilent High Sensitivity DNA kit, where a distinct peak spanning 400 bp to 10,000 bp was observed for all samples, but absent from a parallel negative control run. 1 ng of the resulting cDNA was taken for RNA-seq library generation using the Nextera XT DNA library preparation kit (Illumina). Following tagmentation, each sample was amplified using unique and compatible Nextera XT indexed primers. Samples were then cleaned and size selected using AMPure XP beads, and the yield and size distribution of each amplified cDNA library were assessed using the Agilent High Sensitivity DNA kit with the Agilent 2100 Bioanalyzer. Libraries were then pooled at equimolar concentrations, and sequenced on an Illumina HiSeq3000.

### Transcript assembly and identification

Reads from male and female worms were trimmed to remove adapter sequences using Cutadapt (v.1.8.3) [17]. Trimmed reads were assembled separately *de novo* using Trinity (version 2.2.0) [18]. Reads were mapped back to the respective *de novo* assemblies using Bowtie and the RSEM option in Trinity, to estimate the relative abundance of transcripts. Expression was normalised by calculating fragments per kilobase per million mapped reads (FPKM; [19]). The assembled transcripts were annotated by searching against the *S*. *mansoni* genome, v5 gene predictions, using BLASTn with the default parameters (expect value threshold of less than 10), as the most comprehensively studied and closely related species.

### Predicted properties of head-enriched proteins

The identity of protein products of unannotated genes enriched in the head preparation was sought using BLASTp against the NCBInr database. The properties of the complete subset of proteins potentially enriched in the head (signal peptide, transmembrane helixes, N and O glycosylation sites) was determined as previously described [13]. Potential MEG candidates were assigned based on similarity at the protein sequence level with previously described MEGs from *S*. *mansoni* [13, 20]. Further confirmation of the presence of micro-exons was obtained by examining the gene structure, based on alignment of the transcript sequence with the corresponding *S*. *japonicum* genome segment, using the Splign program available at the NCBI website. Known and putative MEG products were assessed for the presence of highly disordered regions, secondary structure and a long stretch of amino acids (n >16) with a high probability for alpha-helix formation that could indicate the presence of an uninterrupted hydrophobic face, as previously reported [13]. Sequence homologues for conserved domains believed to contain putative binding motifs were sought using hidden Markov models (HMMER v3.1b2 at hmmer.org).

### Molecular phylogeny

Phylogenetic analysis were performed using MrBayes program v3.2.2 [21] with default parameters (4 markov chains) except for the use of the command prset aamodelpr = mixed, which enables sampling across all fixed amino acid rate matrices (models for amino acid evolution) implemented in the program. Analysis were carried out for 1,000,000 generations and the first 250,000 were discarded for production of the trees using the command sumt burnin = 2500. Analysis of MEG-8 and 9 proteins considered 19 unambiguously aligned sites and Jones model was favoured (0.62 posterior probability). Analysis of MEG-26 proteins considered 35 unambiguously aligned sites, with Cprev model favoured (0.91 posterior probability).

## Results

### A subset of genes is differentially expressed in the male heads

**Fig 1:**
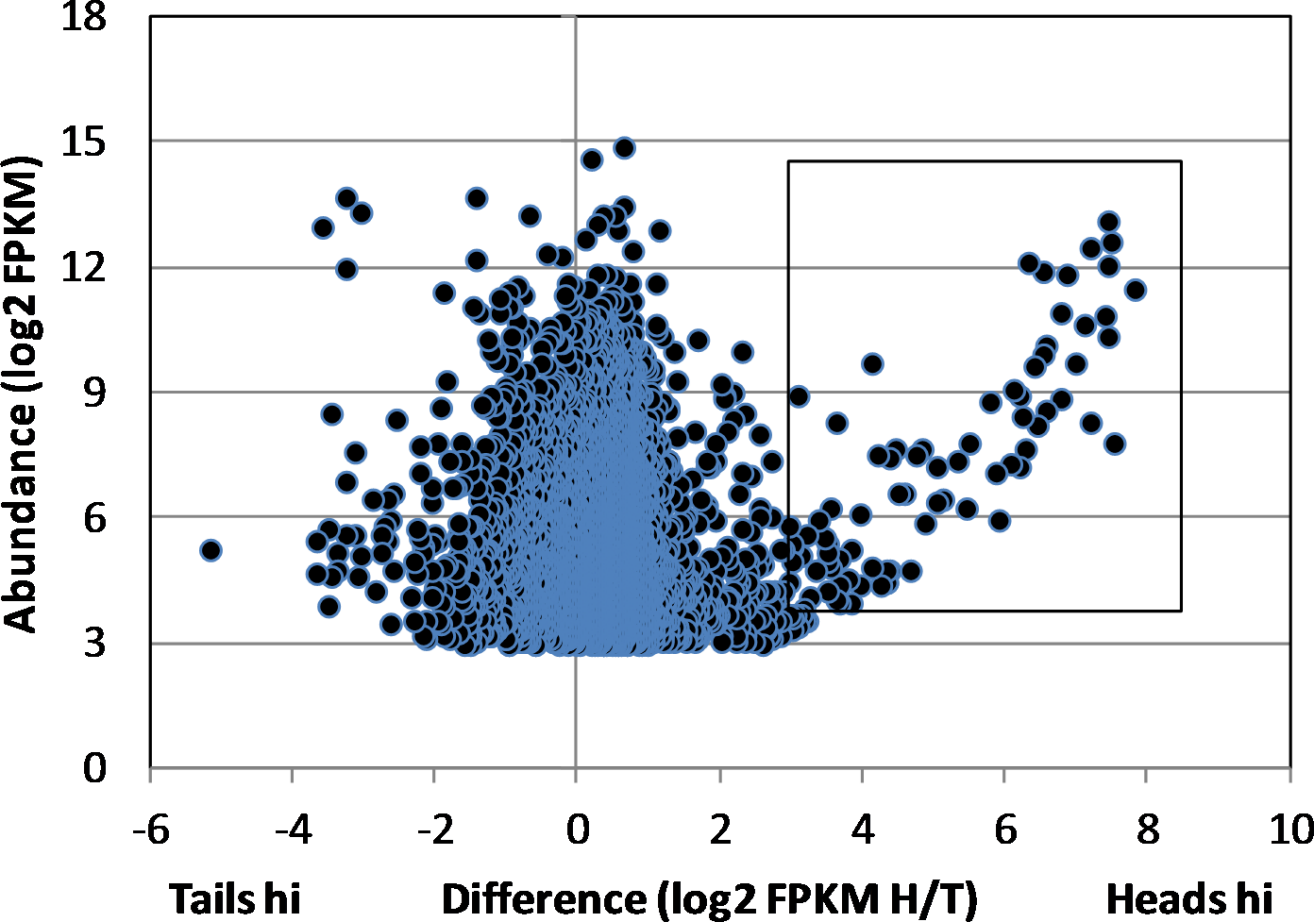
Head-enriched genes in male worms. Scatter plot of differential gene expression in heads and tails based on the Trinity assembly of raw reads by MiSeq. Difference is defined as the ratio of head FPKM/tail FPKM and abundance as the FPKM for each gene. The box delimits the genes that are differentially expressed >8-fold in the heads with a FPKM of >16. Genes with an FPKM <8 are omitted from the dot plot.

Samples comprising 550 ng total RNA were recovered from the male *S*. *japonicum* head homogenate (RNA Integrity Number, RIN = 5.9) and 500 ng from the tails (RIN = 6.1). A 200ng aliquot of each was used to prepare the mRNA library for MiSeq sequencing. This yielded 8 and 12 million 250 bp paired-end reads, respectively. The *de novo* assembly of the combined head and tail reads using the Trinity platform generated >50,000 models, many comprising low read numbers. Elimination of these produced a core of 9204 genes expressed in heads and/or tails with numbers of mapped reads ranging over five orders of magnitude. Of these, 8699 orthologues could be putatively identified in *S*. *mansoni*, by BLAST against the genome database. (N.B. many of these identities are simply ‘hypothetical protein’, which gives no clue to function.) Transcript abundance for each gene in heads and tails was normalised for sequencing depth and gene length by estimating the FPKM and the level of expression in heads and tails compared via the FPKM ratio. This revealed that the expression level of the vast majority of genes varied less than fourfold either side of the equivalence line (Fig 1). Nevertheless, more genes were expressed at a higher level in the heads (6940 genes) than the tails (2263 genes).

The most notable feature of the scatter plot was the group of 84 genes showing a greater than eightfold higher level of expression in the heads (Fig 1, box). A much smaller group of 19 genes was similarly expressed more than eightfold higher in the tails; these had no particular unifying features and will not be further described. Another 22 Trinity gene models were excluded from the detailed heads analysis as they lacked homology to annotated *S*. *mansoni* genes or *S*. *japonicum* cDNAs and ESTs deposited at NCBI. Although the preparation of RNA for sequencing included a polyA enrichment step, it is possible that these excluded assemblies represented non-coding RNA (ncRNA). The number of mapped reads in the remaining heads-hi group of 62 genes ranged from 7.9 x 10^1^ to 1.1 x 10^5^, with a highly skewed frequency distribution (mean 1.41 x 10^4^; median 2.85 x 10^3^) revealing that expression of a small number of genes (~15) was dominant in this tissue.

### MEGs are the major group of differentially expressed genes and encode secreted or membrane proteins

**Fig 2:**
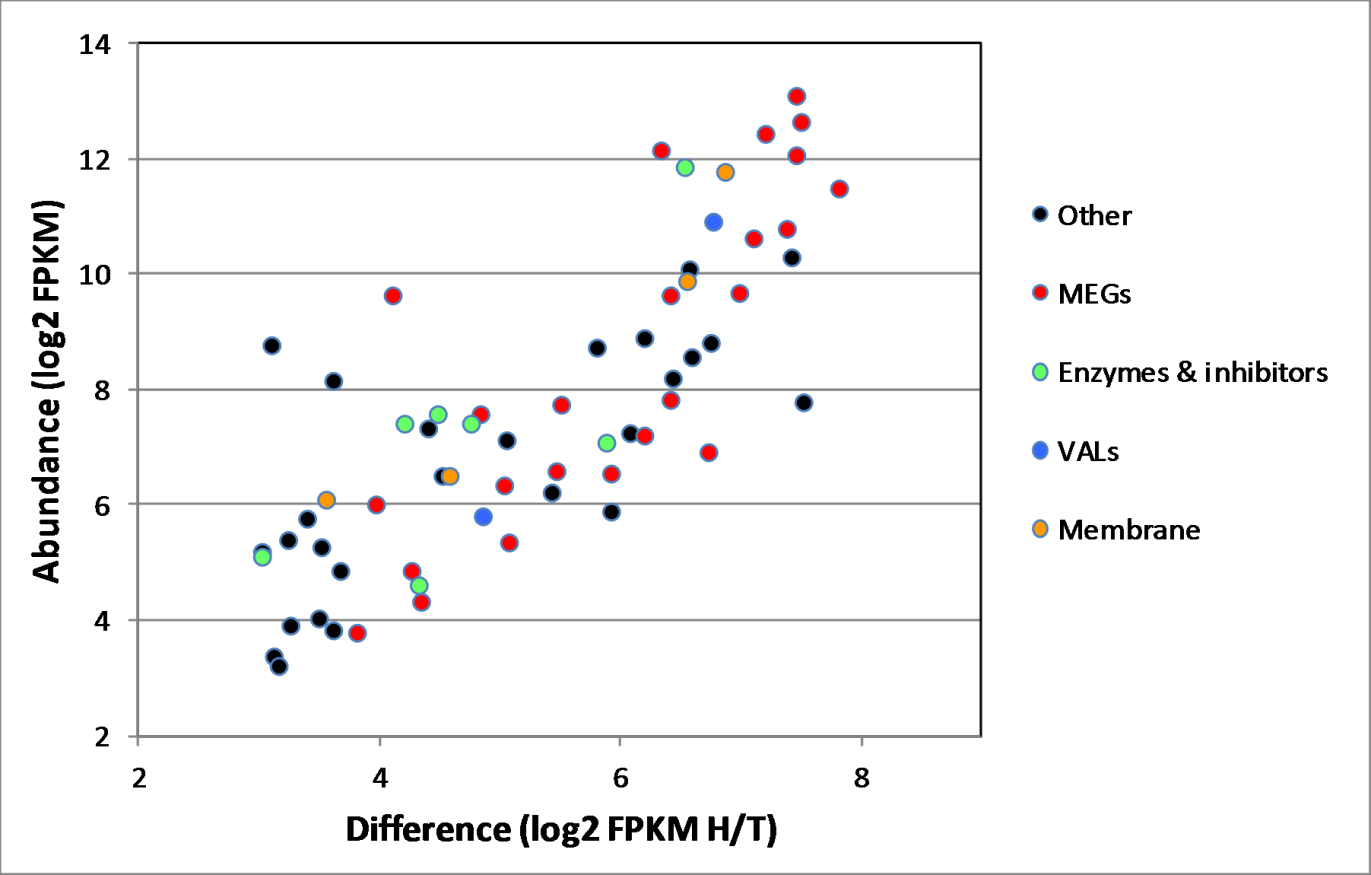
Putative functions of head-enriched genes. Differentially expressed head genes in the box in Fig 1, classified by category.

Analysis of the 62 genes differentially expressed in the head preparation revealed that 24 (> one third) were encoded by MEGs (Fig 2; Table 1). The *S*. *japonicum* MEGs are numbered by their homology with *S*. *mansoni* but there are some gaps as not all those found in the latter species were detected (e.g. MEGs 16 and 17). Taking the FPKM value of each transcript as the normalised measure of abundance, just eight of the MEGs accounted for 90% of the total number of MEG transcripts in the head sample. MEGs also accounted for 11 of the top 20 most highly expressed genes in the differential subset. All the MEGs are predicted by SignalP to encode a signal peptide and three (MEGs 11, 14 and 32.1) are predicted by HMMTop to possess a membrane anchor. The proteins encoded by the remaining 21 are therefore likely to be secreted into the esophageal lumen. NetOGlyc predicts that the three MEG proteins with a membrane anchor are heavily O-glycosylated (5+ sites), as are secreted MEG proteins 4.1, 8.1, 8.3, 15, 22 and 29, suggesting that they may have viscous, mucin-like properties.

**Table 1:** Genes differentially expressed in male heads encoding secretory proteins.

							Log2	
Protein	Trinity			Signal	O/N	Head	Tail	ratio
category	annotation	Sj annotation	Sm homolog	peptide	Glyco	FPKM	FPKM	H/T
MEGs	h12749_g2	SjMEG-8.2	Smp_172180	Yes	O	13.1	5.6	7.5
	h14038_g1	SjMEG-4.2	Smp_085840	Yes	_	12.6	5.1	7.5
	h15148_g1	SjMEG-4.1	Smp_163630	Yes	O	12.4	5.2	7.2
	h15444_g1	SjMEG-14	Smp_124000	Yes	O, N	12.1	5.8	6.3
	h13184_g1	SjMEG-9	Smp_125320	Yes	_	12.1	4.6	7.5
	h12251_g1	SjMEG-29	Smp_243770	Yes	_	11.5	3.7	7.8
	h12430_g1	SjMEG-15	Smp_010550	Yes	O	10.8	3.4	7.4
	h9981_g1	SjMEG-11	Smp_176020	Yes	_	10.6	3.5	7.1
	h14702_g1	SjMEG-12	Smp_152630	Yes	_	9.7	2.7	7.0
	h16155_g1	SjMEG-n.2		Yes	O	9.6	3.2	6.4
	h14159_g1	SjMEG-8.1	Smp_171190	Yes	O	9.6	5.5	4.1
	e106652_g1	SjMEG-26.4		Yes	N	7.8	1.4	6.4
	h7049_g1	SjMEG-8.3		Yes	O	7.8	2.3	5.5
	h10112_g1	SjMEG-8.4		Yes	_	7.6	2.7	4.8
	h14719_g1	SjMEG-26.2		Yes	_	7.2	1.0	6.2
	e103685_g1	SjMEG-26.6		Yes	_	6.9	0.2	6.7
	e104245_g1	SjMEG-26.5		Yes	_	6.6	1.1	5.5
	e80567_g1	SjMEG-n.1		Yes	O	6.5	0.6	5.9
	h14391_g1	SjMEG-19		Yes	O, N	6.3	1.3	5.0
	h17692_g1	SjMEG-26.1	Smp_243740	Yes	_	6.0	2.0	4.0
	e4049_g1	SjMEG-26.7		Yes	_	5.4	0.3	5.1
	e31382_g1	SjMEG-26.3		Yes	_	4.9	0.6	4.3
	h15728_g1	SjMEG-22		Yes	O, N	4.3	0.0	4.3
	h11676_g1	SjMEG-32.1	Smp_123100	Yes	O	3.8	0.0	3.8
Enzymes	h15734_g1	Cystatin	Smp_034420	Yes	O, N	11.9	5.3	6.5
and	h15205_g1	RNAse **Ω** 1	Smp_158430	Yes	O, N	7.6	3.1	4.5
inhibitors	h18246_g1	Asp protease	Smp_132470	Yes	O, N	7.4	2.7	4.8
	h11509_g1	PP HS-esterase	Smp_142970	Yes	N	7.4	3.2	4.2
	h12510_g1	Serpin	Smp_090080	No	O	7.1	1.2	5.9
	h14487_g1	Phospholipase	Smp_031180	Yes	O, N	5.1	2.1	3.0
	h14895_g1	Phospholipase	Smp_031190	Yes	N	4.6	0.3	4.3
VALs	h8808_g1	SjVAL-7	Smp_070240	Yes	O, N	10.9	4.1	6.8
	h15964_g1	SjVAL-13	Smp_124060	No	O	5.8	1.0	4.9
Membrane	h18274_g1	Annexin	Smp_077880	No	O, N	11.8	4.9	6.9
structure	h13936_g1	Annexin	Smp_201250	No	O, N	9.9	3.3	6.5
	h16530_g1	Tetraspanin	Smp_140130	?	N	6.5	1.9	4.6
	h15899_g1	Tetraspanin	Smp_131840	Yes	N	6.1	2.6	3.6
Other	h12339_g1	Natterin	Smp_083240	?	_	10.3	2.9	7.4

### MEGs have an ancient origin that may be related to blood feeding

**Fig 3:**
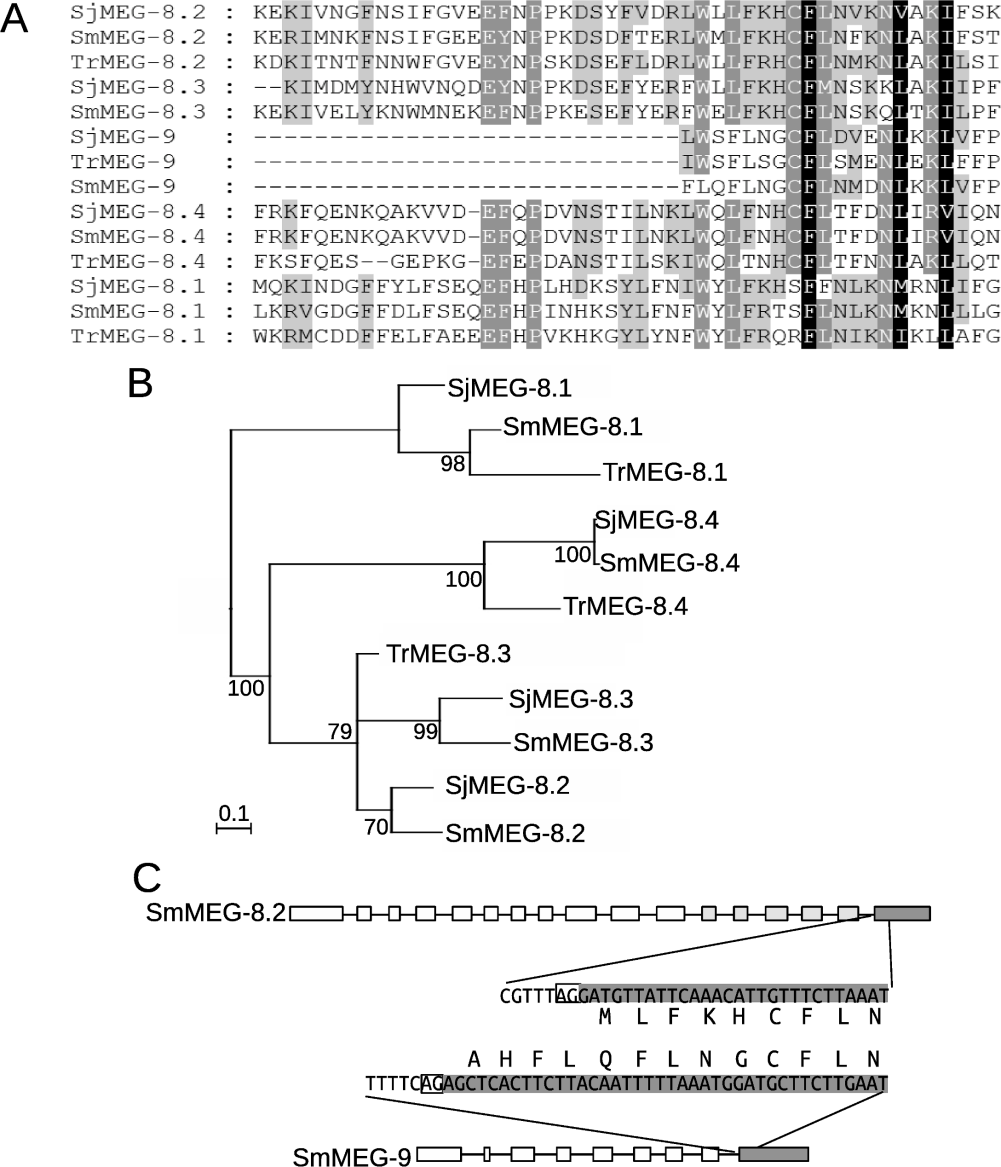
Evolution of MEG-8/-9 family. **A)** Multiple alignment of the residues of the sequence from MEG-8 and MEG-9 family members that displayed detectable similarity at the amino acid level. **B)** Phylogenetic tree of MEG-8 family members based on alignment of the conserved region. The tree was constructed using Bayesian inference implemented on MrBayes. Numbers next to each node indicate posterior probabilities. Nodes with posterior probability lower than 50% were collapsed and the tree was rooted at midpoint. **C)** Schematic representation of gene structure of a representative gene member of the MEG-8 and MEG-9 families. Exon boxes are proportional to their lengths in bp. Lines represent introns shown with a length not proportional to their size. Boxes in light and dark grey indicate exons coding for residues conserved in MEG-8 or in MEG-8 and MEG-9 families, respectively. The nucleotide and protein sequence at the 5’ boundary of the last exon and the adjacent intron is shown, with homologous residues aligned. The white box in the nucleotide sequence highlights the canonical splicing site.

The presence of a conserved hydrophobic C-terminal protein domain containing a putative binding motif was previously described in the two MEG-4 family members, across three *Schistosoma* species [7]. Analysis of transcriptome assembly data from the bird schistosome *Trichobilharzia regenti* [22] identified orthologs of MEG-4 displaying a similar structure (S2 Fig). A hydrophobic C-terminal domain with a distinct motif was also previously reported in the four MEG-8 family members [13]. Interrogation of the sequence database for *T*. *regenti* returns significant matches to SjMEGs 8.1, 8.2 and 8.4 (Fig 3A). Phylogenetic analysis suggests that the gene duplications originating MEG-8.1 and -8.4 were an early event in the evolution of the Schistosomatidae (Fig 3B). A more convoluted scenario occurs for the branch containing MEG-8.2 and -8.3, where the lack of a *T*. *regenti* equivalent to the latter gene and ambiguous tree topology (Fig 3B) makes the timing of gene duplication uncertain. Searching of *S*. *mansoni* proteins v4 on GeneDB, using HMMER with an HMM based on an alignment of the conserved C-terminal domain of MEG-8 family members, also returns a hitherto unsuspected but significant match (e-value 0.0088) with SmMEG-9. Extending this approach, comparison of the amino acid sequences of SjMEGs 8.1 to 8.4 and SjMEG-9 using HMMER again reveals a common motif. Furthermore, *T*. *regenti* possesses a MEG-9 gene that encodes a single copy of the conserved motif in its C terminal domain. It is notable that practically all residues detected as conserved between MEG-8 family members and MEG-9 are coded by the C-terminal exon, which in both instances can be classified as phase 2 at its 5’ extremity, as the first base of the exon is the third base of a codon (Fig 3C). It is noticeable, however, that the MEG-9 terminal exon is a little longer than MEG-8 family members at its 5’ end.

### Many MEG proteins are predicted to have amphipathic properties

The presence of an amphipathic helix with a hydrophobic interaction face was previously noted in *S*. *mansoni* MEG-12, localised to the anterior esophagus [13]. Heliquest similarly predicts that *S*. *japonicum* MEG-12 and the MEG-26 family (see below) possess amphipathic helices with a hydrophobic moment >0.4 and a net charge >3/-3. MEGs 9 and 19 are also predicted to have a hydrophobic interaction face but with a lower hydrophobic moment, namely a net charge of 0 and +1, respectively (S3 Fig). Conservation of the character (charged, polar or apolar) of the amino acid residues for most of the positions, when comparing *S*. *japonicum* and *S*. *mansoni* sequences, is suggestive of a complex mechanism of interaction with membranes rather than a solely hydrophobic-driven effect. The amphipathic face of MEG-9 partially overlaps with one copy of the conserved motif, suggesting a dual capacity for host cell interaction via a hydrophobic lipid bilayer and more specifically with a potential protein receptor. Although neither MEG-15 nor MEG-29 is predicted to have an amphipathic helix, both possess a hydrophobic C-terminal region balanced by a group of predicted hydrophilic N-terminal O-glycosylation sites.

### The MEG-26 genes encode a family of proteins with amphipathic helices that have diversified during schistosome evolution

**Fig 4:**
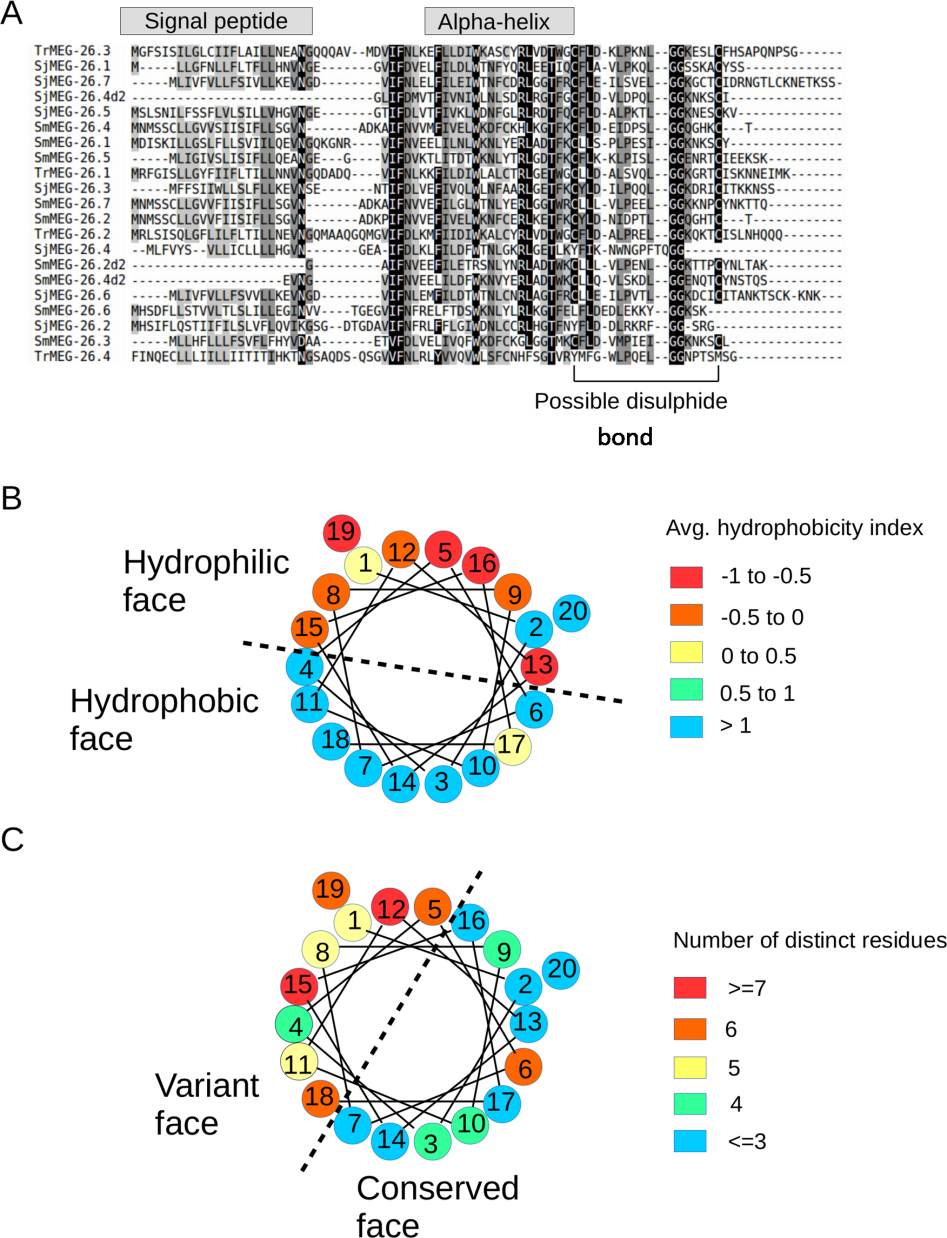
Analysis of the protein sequence of MEG-26 family numbers. **A)** Multiple alignment of the MEG-26 families from *S*. *japonicum*, *S*. *mansoni* and *T*. *regenti*. The repeated domains of SmMEG-26.2 and -26.4 and SjMEG-26.4 are separately aligned and given a “d2” suffix. Grey rectangles above the alignments indicate predicted regions for the signal peptide and the amphipathic alpha helix. **B and C** Helical wheel representation of the amphipathic helix with numbers representing the sequential order in the primary sequence. Colours in **B** represent the average hydrophobicity considering all members in each position of the helix, using the values from a previously determined hydrophobic parameter [23] and in **C)** the numbers of different residues in each position taking all family members into consideration. Dashed lines indicate separation between faces of the helix having different polarity or conservation of residue properties.

A single MEG-26 gene was originally described as expressed in the head region of male *S*. *mansoni* worms [13]. Using the transcript data from the *S*. *japonicum* head preparation, we have expanded the analysis to identify a closely related family of MEG-26 genes with seven members (Fig 4). Interrogation of *S*. *mansoni* and *T*. *regenti* databases with these SjMEG-26 family transcripts using TBLASTN, permitted the description of seven and three additional family members, respectively, in the two species. Alignment of family member protein sequences highlighted those residues with a high degree of conservation (Fig 4A). It is notable that two genes from *S*. *mansoni* (SmMEG-26.2 and 26.4) and one from *S*. *japonicum* (SjMEG-26.4) display a tandem repetition of a conserved protein motif, thus suggesting the occurrence of an internal duplication (Fig 4A and S4 Fig). Indeed, examination of the gene structure for *S*. *mansoni* reveals the repetition of an identical exon length pattern. Analysis of the phylogenetic tree for the MEG-26 family suggests several species-specific recent events of gene duplication (S5 Fig), indicating the presence of evolutionary pressure for diversification of sequences. It is notable that all MEG-26 transcripts encode a signal peptide and a region with a predicted amphipathic helix (Fig 4A). Analysis of these helix sequences from all MEG-26 members clearly shows distinct hydrophobic and hydrophilic faces (Fig 4B). Inspection of amino acid conservation also shows distinctive conserved and variable faces of the helix (Fig 4C).

### Hydrolases, protease inhibitors, VALs and membrane structural proteins are differentially expressed in the male heads

Transcripts encoding a group of five hydrolases were identified in the head preparation, namely Ribonuclease T2, Aspartyl protease, Palmitoyl protein thioesterase 1 and two Phospholipases (Fig 2, Table 1). All possess a signal peptide and are usually associated with lysosomes, having an acidic pH optimum. Transcripts for two protease inhibitors, a serpin and a cystatin, were found; the encoded proteins function to inhibit serine and cysteine proteases, respectively. The cystatin transcript was the sixth most abundant and encoded a signal peptide whereas the serpin did not. Two representatives of the venom allergen-like gene family were enriched in the male heads, group 1 member VAL-7 (secreted) being abundant and group 2 member VAL-13 (cytosol) much less so. Transcripts encoding four membrane-associated proteins, two annexins and two tetraspanins, were also enriched in the heads, and it is plausible that these are expressed on the esophageal lining. The final abundant transcript of note encoded a domain of unknown function, DUF3421, found in the protein constituents of toadfish (*Thalassophryne nattereri*) venoms and termed Natterins. It is ambiguous whether the *S*. *japonicum* gene encodes a signal peptide but the fish natterins are secreted. That leaves approximately 20 miscellaneous genes enriched in the heads, six of which encode proteins with a signal peptide so could be secreted or membrane-associated. Four of these have no other distinguishing feature, one is a glycosyl transferase and the last a glucose transporter (S2 Table). This transporter is a 12-spanning membrane protein with nearest homology to the solute carrier family 2, facilitated glucose transporter member 8, also known as GLUT8.

### Similar patterns of differential gene expression are found in the female head region

**Fig 5:**
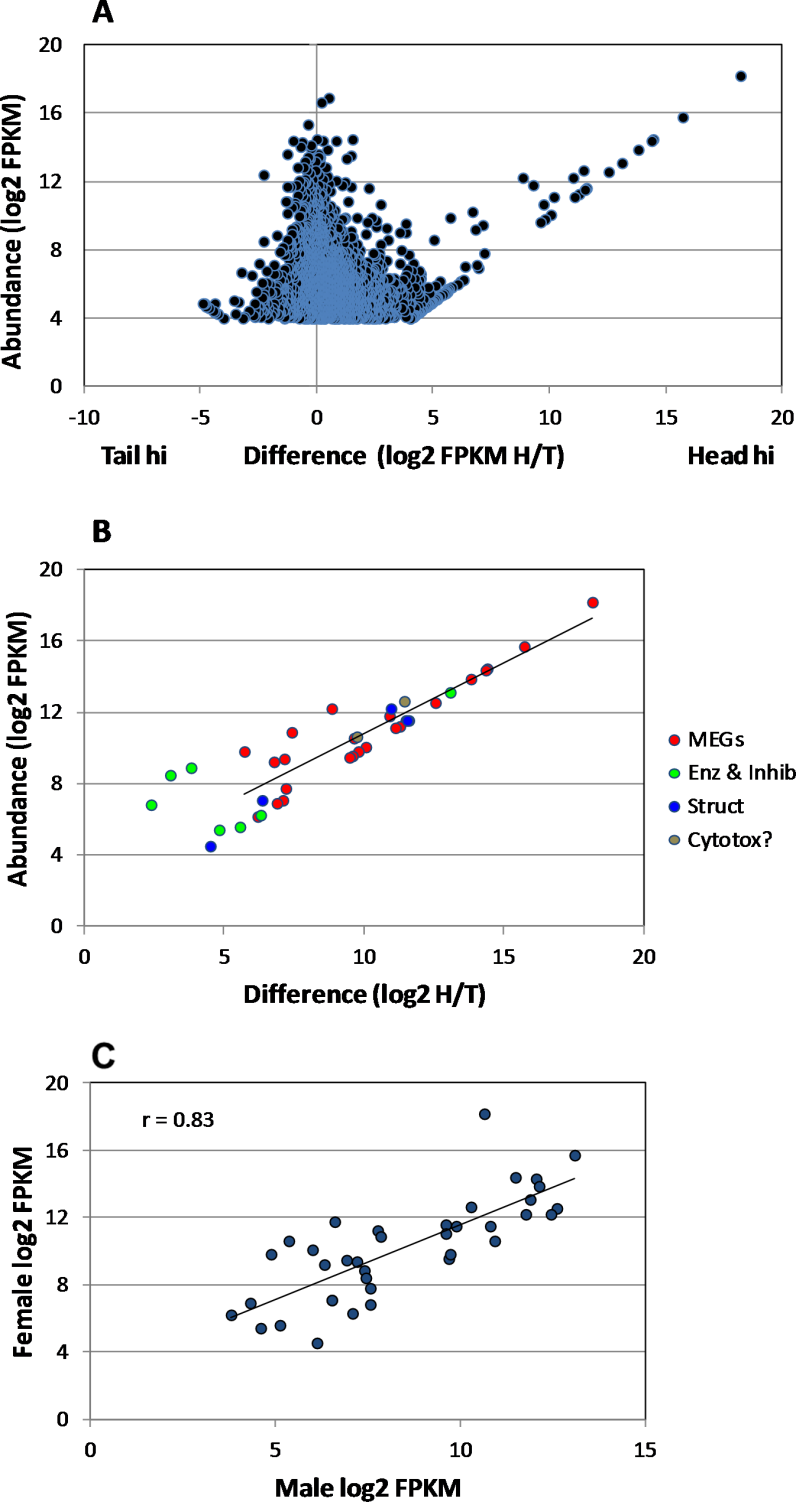
Head-enriched genes in female worms. **A)** Scatter plot of differential gene expression in female heads and tails, based on the Trinity assembly of raw reads from HiSeq. Those genes differentially expressed >16-fold in the heads with a FPKM of >16 were considered for analysis. B) Differentially expressed genes encoding secreted or membrane proteins classified by category. C) A comparison of the expression level of selected genes in male and female heads. The correlation coefficient r between the two parameters = 0.83.

Precise excision of female *S*. *japonicum* heads was technically difficult due to their small size and the preparation obtained yielded 17.6 ng total RNA (RIN = 5.9); recovery from the female tails was not limiting (RIN = 6.2). HiSeq sequencing of the female libraries, prepared using the SMARTseq kit, yielded 13.3 and 19.6 million paired-end reads for the heads and tails, respectively. *De novo* assembly of the combined female head and tail reads generated a large number of gene models, which reduced to 5429 genes expressed in heads and/or tails, on the basis of similarity to *S*. *mansoni* genes, with novel MEG sequences from male *S*. *japonicum* heads added, to ensure continuity in male-female comparisons. Elimination of low abundance transcripts (FPKM <16) provided a core of 3749 genes for analysis. As with the males, more genes were expressed at a higher level in heads (2434) than tails (1315) (Fig 5A). The scatter plot revealed a group of 224 genes expressed at eightfold or higher level in the heads. The most highly expressed paralleled those in the male head, with 25 MEGs, 6 enzymes and inhibitors, 4 membrane structural proteins, plus SjDUF-Natterin and SjVAL-7 (Fig 5B; S2 and S3 Tables). A gender comparison of levels of expression for the 38 highlighted genes reveals a correlation coefficient r = 0.83 (Fig 5C). SjMEG-11 was the marked outlier with much greater expression in the female than male. We make no inferences from the higher log2 FPKM of the selected group in females (mean = 10.2) than in males (mean = 8.37) (S2 and S3Tables; Table 1). This could reflect the techniques of library construction and sequencing as much as gender differentials in gene expression. Considering group expression levels within the female sample, most of the MEG transcripts that top the list in males also predominate in females, implying no obvious gender bias in esophageal functions.

Apart from SjCystatin, as in males the sixth most abundant transcript, the enzymes and serpin are expressed at one thirtieth the level of the MEGs. Given similar rates of translation, this could reflect the fact the hydrolases react with multiple substrate molecules, whereas MEG proteins may be capable of only a single binding interaction with the target. The same is true of the SjVAL-7 and DUF-Natterin proteins where transcript levels are also higher than for the hydrolases (Fig 5B; S2 and S3 Table). Although no individual MEG-26 gene is expressed at a high level in the female head, the cumulative total of FPKMS for all family members (= 10487) places them as the 6th most abundant esophageal transcript; this implies an important role in blood processing for the protein products. In comparison, the same cumulative total in male heads (= 733) places the family as the 17^th^ most abundant.

## Discussion

MEGs, first reported in 2009 for *S*. *mansoni* [24], are an enigmatic group of genes with ~80% of the protein coding region comprising short symmetric exons ranging from 6 to 36 bp; they appear to represent a novel system for generating protein variation by alternative splicing [20]. Almost without exception, MEGs encode secreted or exposed membrane-anchored proteins and production of protein variants was confirmed by proteomic analysis of the secretions of migrating intravascular schistosomula (MEG-3 family) and mature eggs (MEG-2 and MEG-3 families) [20, 25]. Initially, upregulation of several MEG genes was detected in Day 3 schistosomula by DNA array analysis, and shown to be localised to esophageal gland primordia [26]. Use of subtractive RNA-Seq, combined with WISH, revealed that the esophageal glands of adult male *S*. *mansoni* were a hotspot for expression of at least 27 MEGs, while the total number of MEG families was increased to 32 [13]. A second minor assemblage comprising MEGs 1, 5, 6, 13 and 24 was putatively assigned to the tegument, MEG-5 having been previously identified in tegument fractions by proteomics (S6 Table in [20]). The overall picture is thus of a group of genes with unusual structure that encode proteins secreted from glands or epithelial surfaces of larvae, adult worms and eggs that have no orthologies with known proteins. Being secreted or surface-exposed, all must come into contact with the host immune system and it is tempting to conclude that the production of protein variants is part of an immune evasion strategy at the critical parasite-host interface.

### MEGs in the *S*. *japonicum* esophagus

The SjMEG-4.1 protein was previously localised to the posterior esophageal gland by immunocytochemistry [7]. Subsequently, we identified SjMEGs 4.2, 8.2, 9, 11 and 14 by homology searching and showed by immunocytochemistry that their proteins were present in the same gland [15]. Their co-localisation with host IgG in the esophageal lumen of surviving worms recovered from self-curing rhesus macaques led us to suggest that they might represent novel vaccine candidates [15]. We have therefore deployed the subtractive RNA-Seq approach to compile an inventory of esophageal gland MEGs and other secreted proteins in *S*. *japonicum* as a prelude to investigating their immunoreactivity and vaccine potential in the rhesus macaque model.

RNA-Seq applied to male *S*. *japonicum* heads and tails greatly expands the previous total of six MEGs, bringing the total number present in the *S*. *japonicum* genome to approximately 30 and revealing that these enigmatic genes are an early feature of the Genus. Not all counterparts of the *S*. *mansoni* MEGs were found. This could simply reflect the previously described rapid evolutionary changes that have occurred in some MEGs within the Genus to render homology searches ineffective [16]. On the other hand, a more complex scenario could also involve deletion events and creation of novel MEGs from duplication and shuffling of existing micro-exons, generating mosaic proteins containing micro-regions of homology, which would not be easily detectable by alignment methods. Any of these scenarios appear to highlight the fact that MEGs represent a very dynamic system and the repertoire of MEGs could be highly influenced by the host range of each species.

It is of note that we were able to identify orthologues of MEG-4 and MEG-8 families, plus MEG-9 in the bird schistosome *T*. *regenti*. This discovery in a separate Genus of blood-flukes, parasitizing a different order of the Phylum Chordata, but not in transcript databases of other trematode families such as the Fasciolidae or Opisthorchidae, or in cestodes, reinforces their ancient origins and also the notion that esophageal MEG proteins have specific functions related to blood feeding.

The previously described hot spot for MEG expression [13] in the esophageal glands of *S*. *mansoni* is now replicated in *S*. *japonicum*. Indeed, it is plausible that, in spite of their small size, the esophageal glands are among the most biosynthetically active tissues in the adult worm. The FPKM values (normalised transcript abundance) of the head MEGs compare with those of the genes encoding the cathepsin products of the gastrodermis, providing evidence for this suggestion. Our ultrastructural and immunocytochemical observations have revealed that erythrocyte lysis, leukocyte tethering and death all occur in the esophagus lumen. The transcript dataset from both male and female worms contained appreciable numbers of sequences from the rabbit host, originating in the leucocytes ingested during blood feeding, which have been analysed separately. Systematic analysis of the pathways revealed by the rabbit transcripts points to a plausible sequence of events that occur in the ingested cells, especially the neutrophils and monocytes, which find themselves in a foreign environment. As might be expected there is evidence for the triggering of both inflammatory and innate immune responses in the leucocytes. Genes known to be involved in Fcγ-mediated phagocytosis and lysosomal pathways of antigen processing are also actively transcribed. This burst of aggressive activity appears to be short-lived as genes linked to pro-apoptosis are active, suggesting that the effector cells rapidly succumb to apoptosis. The time frame for these events is likely to be only minutes. Both the structural and gene expression data point to a battle between the worm and the host fought in the esophageal lumen, which the host normally wins. They provide a context for the multiplicity of secreted proteins released from the glands and a major task is to match them to the biological processes. To date, only the interaction of MEG-14 with neutrophil inflammatory protein S100 has been demonstrated experimentally in *S*. *mansoni* [27]. Flow cytometry would be a plausible way to investigate the interaction between MEGs and blood components such as leukocytes but the hydrophobic nature of many MEGs has so far thwarted our efforts. We must therefore currently rely on a bioinformatic analysis of the properties of MEG proteins to infer functions.

### Interaction with host cells: Putative leukocyte binding motifs

A subset of MEG proteins possesses clearly identifiable shared protein-binding motifs, as previously described for the MEG-4.1 and MEG-4.2 family members in *S*. *mansoni* and *S*. *japonicum* (S6 Fig in [7]). It now appears that the two MEG-4 genes had already diverged in the bird schistosome *T*. *regenti*, with the common motif residing in the long hydrophobic C terminal amino acid sequence. The MEG-4 proteins are specifically associated with the exterior of ingested host leukocytes in the esophagus lumen [7], suggesting that the motif provides a mechanism for interaction with their surface components. The MEG-8 family also possesses a common motif and binds to cells *in situ* in the esophageal lumen of *S*. *mansoni* and *S*. *japonicum*. We have traced the MEG-8 motif back to *T*. *regenti* where three members are present. Extension of these searches using hidden Markov model-based tools (HMMER) also showed that the common MEG-8 motif was encoded by the C-terminal exon of MEG-9, even though the remainder of the two genes do not encode similar protein sequences. A plausible explanation is that an ancestral recombination event occurred where the original terminal exon of MEG-9 was substituted for a MEG-8 exon (or vice-versa).

Of note, the remainder of the MEG-9 protein has an amphipathic helical structure placing it in the other major subgroup of MEGs (see below), and so it may interact with both a protein ligand and the lipid bilayer of a target cell. The conserved variants of the MEG-4 and MEG-8/9 motifs may be important for interaction with groups of structurally related receptors on the surface of ingested host cells but their protein binding partners have yet to be identified.

### Interaction with host cells: Amphipathic properties

The second major group of MEG proteins secreted from the esophageal glands possesses an amphipathic helix conformation that may facilitate interaction with the (hydrophobic) lipid bilayers of host cells. This feature is defined as the segregation of hydrophobic and polar residues between the two opposite faces of the α-helix, a distribution well suited for membrane binding [28]. Notably, several of the *S*. *japonicum* helices have a cationic character, similar to that observed for amphipathic helices from antimicrobial peptides, which facilitate interaction with negatively charged outer membrane surfaces [29]. In addition to MEG-9, the proteins include MEG-12, MEG-19 and the MEG-26 family. Again, the multiplication of MEG-26 genes in both *S*. *mansoni* and *S*. *japonicum* may be the result of high evolutionary pressure by the host immune system [16]. The outcome is the secretion into the esophageal lumen of a mixture of short helical peptides with hydrophilic and hydrophobic faces (too short to take on a globular conformation). In these amphipathic proteins it is plausible that the helix face with lower conservation represents the residues exposed to the environment, while the most conserved face contains buried residues not exposed to the host immune system. The fact that the hydrophilic face is not totally coincident with the variable face may indicate that MEG-26 interactions are not driven solely by a hydrophobic effect, e.g. with a generic lipid bilayer, but could also involve a specific component, i.e. a protein or glycan motif. There is independent experimental evidence for the concept that proteins with amphipathic helices (FhHDM-1 and Sm16) secreted by digenetic trematodes, can interact with host leukocytes, and modulate the host immune response [30, 31]. There are several members of the Sm16/HDM family in *S*. *japonicum*, *S*. *mansoni* and *T*. *regenti* and it is tempting to hypothesize that since MEG-26 is structurally related to these proteins it might possess analogous properties that aid binding to host leukocytes.

The production of amphipathic secretions by the esophageal glands also provides an explanation for features of their ultrastructure. A large fraction of the matrix of the crystalloid vesicle, the major secretory inclusion of the posterior gland, has a characteristic pseudocrystalline appearance [7]. Originally interpreted as parallel layers of membrane [5] we concluded that they were quasi-molecular structures of highly ordered proteins/glycoproteins that self-assembled after packaging in the Golgi apparatus of the gland cell bodies. The contained parallel array of material is released intact to the esophageal lumen, at specific docking sites, by fusion of the vesicle membrane with the surface plasma membrane [5, 7]. Thereafter the arrays do not dissipate, as would be expected for normal secretory vesicle contents, but cluster into larger aggregates, thus confirming the self-affinity of their molecular constituents.

### Hydrophobicity problem

The hydrophobicity of part or all of numerous MEG proteins very likely determines their propensity to aggregate within secretory vesicles and between the plates that line the posterior esophagus, to form the observed pseudocrystalline structures. The hydrophobic face would primarily serve the purpose of promoting the self-assembly of the arrays (molecular aggregates) seen in the electron microscope. It seems unlikely that after secretion, these proteins would be freely and readily soluble. Instead, their hydrophobic faces would further interact in the spaces between plates, creating the larger aggregates for ‘storage’ of MEG proteins. These large aggregates would decrease in size upon close contact with passing cells, when the active monomers partitioned into the hydrophobic membrane environment. The “storage” of released secretions between plates would also prevent their wash-out (and waste) when worms vomited their residual gut contents.

### Presence and function of non-MEG secreted proteins

Among the oesophageal genes/proteins of male *S*. *mansoni* we reported the presence of VAL-7 [13], which is also a prominent product of the *S*. *japonicum* esophagus. Schistosome VALs are members of CAP or SCP/TAPS protein superfamily Pfam PF00188, present in a wide variety of eukaryotes including parasitic helminths, insects and plants [32]. The crystal structure of several members is known, including SmVAL4 [33] that possesses a single CAP domain. Protein structural conservation within the CAP superfamily results in fundamentally similar functions for the CAP domain while the diversity outside this core region affects the target specificity of each CAP protein [34]. That primary function appears to provide the capacity for binding lipids such as palmitate, cholesterol or leukotrienes [35, 36]. By analogy with SmVAL-4, it seem likely that VAL-7 will also bind lipids but that its interaction with ingested blood proteins or cells will be determined by extrinsic loops and cannot be predicted by amino acid sequence alone [33].

A group of lysosomal hydrolases was also identified, including orthologues of the two phospholipases and palmitoyl protein thioesterase 1 present in *S*. *mansoni*. However, only a single aspartyl protease was detected, suggesting that the six versions found in *S*. *mansoni* are the result of more recent gene duplications, potentially related to its increased host specificity. (*S*. *japonicum* has a wide host range and its clade is considered to be more basal in the phylogenetic tree [14]). The presence of an abundant transcript encoding a domain of unknown function (DM3421) that is shared with a group of venoms termed Natterins is intriguing. The fish Natterins are kininogenases that act on plasma kininogen to release kallidin/bradykinin, causing pain and oedema in the victim [37, 38]. These enzymes are also plasminogen activators, causing activation of plasmin that breaks down fibrin clots. The occurrence of such enzymatic activity in the esophageal secretions could partly explain the absence of blood clotting. Conversely, our observation that host IgG colocalises with fibrin deposits in the anterior esophageal lumen of worms from self-curing rhesus macaques (rhesus paper S4 Fig) suggests that SjNatterin would be a good vaccine target. In parenthesis, it should be noted that in our previous study [13] the *S*. *mansoni* orthologue (Smp_167280) was the 15^th^ most abundant differential transcript in the male heads by RPKM value, but annotated as a hypothetical protein.

Transcripts for two protease inhibitors, a cystatin and a serpin were prominent in both male and female heads; neither was differentially expressed in the *S*. *mansoni* male head preparation [13]. Extensive searching of public databases failed to find a cystatin orthologue in *S*. *mansoni* although candidate CDS were present in the genome scaffolds of *S*. *curassoni*, *S*. *mattheei*, *S*. *margrebowei* and *S*. *rodhaini*. The *S*. *mansoni* serpin orthologue (Smp_090080) has been reported in proteomic analysis of worm vomitus which could be consistent with an esophageal origin. N.B. the *S*. *japonicum* serpin transcript is not identical with one previously described in that species [39]. As there are no serine proteases reported from the schistosome gut (wrong pH optimum?) it is feasible that its inhibitory function is exerted against ingested plasma proteases. These include thrombin, which converts fibrinogen to fibrin during clotting, and the serine proteases of the Complement system. It would be an advantage for the worm to inhibit both clotting and Complement fixation in the narrow confines of the esophagus lumen. The cystatin transcript is highly abundant and the obvious targets for its protein product are the cysteine proteases B1, B2 and L produced by the gastrodermis [1, 2]. Potentially cystatin would allow the protein secretions of the esophageal glands to perform their functions on ingested blood cells and proteins without being destroyed by reflux of the gut proteases. In an analogous situation, both serpins and cystatins are abundant in the saliva of blood feeding ticks, where they have been shown to impair the functions of host leucocytes and inhibit blood clotting (reviewed in [40]).

### Male versus female gene expression in relation to esophageal capabilities

The daily blood consumption of *S*. *mansoni* females is > eight times that of the males [41]. Although never formally measured in *S*. *japonicum*, the discrepancy is likely to be larger given the greater nutrient requirement of the females to support the higher level of egg production. (Visual inspection of the “black” females confirms their much greater haemozoin content than males [2].) This presents a conundrum as the male esophageal glands are about four times the size of those in females yet the female processes much more blood [41]. We had anticipated that differences in esophageal gene expression between the two sexes would provide at least a partial explanation but, with the exception of MEG-11, there was a good association between the transcript levels of esophageal genes in males and females. This appears to rule out regulation of esophageal gland secretions at the level of gene transcription. The most plausible alternative is that secretion is regulated at the level of translation. We previously estimated that the oral and esophageal pumps in the female must work 40 times as hard as those in the male to ingest the requisite volume of blood [2]. Consequently, females must feed continuously, with short interruptions for regurgitation of hemozoin, whereas males feed intermittently. A comparison of rates of synthesis of esophageal proteins in males and females should settle this question.

## Conclusions

It is clear from this and our previous studies that the schistosome esophagus is a complex structure performing multiple functions related to the processing of ingested blood in its anterior and posterior compartments [7, 10, 13]. It is equipped, via the secreted products of the surrounding anterior and posterior glands, with a range of diverse proteins to execute these tasks. MEG proteins are the major secretions and fall into two broad categories, those potentially capable of binding to specific ligands on ingested material and those capable of hydrophobic interactions with lipid bilayers. They are assisted in their actions by lysosomal hydrolases and potential inhibitory molecules. The end result is the lysis of erythrocytes and the disabling of leukocytes, without the formation of a fibrin plug or Complement fixation. These esophageal secretions represent an entirely new category of vaccine candidates and the self-cure mechanism in the rhesus macaque provides the context in which to evaluate their role as mediators of protection. We have already suggested that the slow starvation and death of adult worms in self-curing macaques is the result of blocked feeding mechanisms in the esophagus, and the female is apparently more vulnerable than the male [15]. In a subsequent publication we will report on the immunoreactivity of the esophageal secreted proteins of *S*. *japonicum* in the rhesus macaque model of protective immunity.

## Supporting information

S1 Appendix MEG-26 families, nucleotides and ORFs in *S. mansoni, S. japonicum* and *Trichobilharzia regent*. (DOC)

S1 Fig To scale diagram of male and female *S. japonicum* worms to show the excision points for heads and tails in the two sexes. The shaded area in the female indicates the range of the excision point due to the small size of the female head region.
(PPTX)

S2 Fig Alignment of the MEG-4 C-terminal region of bird and mammal schistosomes showing conservation of the binding motif. (TIF)

S3 Fig Conserved regions of MEGs 9, 12 and 19 in *S. mansoni* (Sm) and *S. japonicum* (Sj) compared. Hydrophobic/amphipathic helix regions within the boxes are denoted HHHH. Helical wheels, drawn using the Heliquest program, display the disposition of residues as follows: apolar = yellow; positively charged = blue; small side chains = grey. The remaining coloured residues represent polar chains. Arrows represent the hydrophobic momentum vector.
(TIF)

S4 Fig Schematic representation of *S. mansoni* MEG-26 family members. Gene structures of SmMEG-26.2 and 26.4, each with two amphipathic helixes, and MEG-26.1 with a single amphipathic helix are shown. Lengths of white exon boxes are proportional to their nucleotide complement in bp. Thin intron lines are not proportional to their size. Thick lines represent portions of the sequenced UTR and are proportional to their size. Numbers above each exon represent the size of the coding region, including stop codons, expressed in base pairs.
(TIF)

S5 Fig Phylogenetic tree of MEG-26 family members, based on their multiple alignments. The repeat domains of SmMEG-26.2 and were considered as an independent sequence and given a “d2” suffix. The tree was constructed using Bayesian inference and implemented on MrBayes. Numbers next to each node indicate posterior probabilities. Nodes with posterior probability lower than 50% were collapsed. The tree was rooted at midpoint.
(TIF)

S1 Table Male worm head and tail gene expression. (XLSX)

S2 Table Female worm head and tail gene expression. (XLSX)

S3 Table Top female head enriched transcripts sorted by abundance (FPKM). (TIF)
