# Supplementary material for: Microexon gene transcriptional profiles and evolution provide insights into blood processing by the Schistosoma japonicum esophagus
Source: PLoS Negl Trop Dis. 2018 Feb 12;12(2):e0006235. doi: 10.1371/journal.pntd.0006235 (PMC5825161; doi:10.1371/journal.pntd.0006235)
Supplement: S1 Appendix — (DOC) [file pntd.0006235.s001.doc]

**MEG-26 families in *Schistosoma mansoni*, *S. japonicum* and *Trichobilharzia regenti***

Signal peptide

>SmMEG26.1_Smp_243740

CGACGGTATATTAGTTAAATAATATGGATATCAGTAAAATTTTACTGGGATCTTTGTTTTTACTTTCAGTAATCATATTACAAGAAGTGAATGGACAAAAGGGAAATAGAGTTATCTTCAATGTCGAAGAGCTTATCTTGAACCTCTGGAAAAACTTGTACGAACGTTTGGCTGACACATTCAAATGTCTTTTGAGCCCATTACCAGAATCTATTGGCGGTAAAAATAAAAGCTGTTACCCTTAGTAACATTCAAAAAGTTTACTTCCTGAAATTTTATAGAAAAAACATCAGCTACAAATAAAAAAATATAACCTTACTGAT

>SmMEG-26.1

MDISKILLGSLFLLSVIILQEVNGQKGNRVIFNVEELILNLWKNLYERLADTFKCLLSPLPESIGGKNKSCYP

>SmMEG26.2

CTGTAACATGGTGTAGTTTTACCTCCCAGATTTTCAGGTAGTACTAGTAATAAGCATTTCCAAGTATCAGCTAAGCGATTGTATAAATTGCTCCGGGTCTCCAAAATAAATTCTTCAACATTGAAAATCGCTCCTGTACATGTATGACCTTGGCCGCCGAGAGTTGGATCAATGTTGTCTAAATAGCATTTGAAAGTTTCCTTCAGTCGTTCGCAGAAATTTTTCCATAGTTCTACAATAAACTCCACGACATTAAAGATTGGTTTATCAGCATTCACTCCACTAAGTAAAAATATTGAAATGATGAATACTACGCCCAACAGACAACTACTCATATTCATGATTATTTATTATCCTCTAAGAAGTAAACTTTGCAGAAACACGTTCAGTAAAATCGTAAAATGCGCGCA

>SmMEG-26.2

MNMSSCLLGVVFIISIFLLSGVNADKPIFNVVEFIVELWKNFCERLKETFKCYLDNIDPTLGGQGHTCTGAIFNVEEFILETRSNLYNRLADTWKCLLLVLPENLGGKTTPCYNLTAK

>SmMEG26.3

CCATCACTACAAAAGCTCAAACTGATCATAATGAAAAGTTATTAATTTCTATGGTCAAATGTAAAATGGTGTGTGAATTCCTATCAAAGAATAAAACATGTAATTAGGTTGCTTTTGAAAATTACAACTTCCATTGTTGCCTAACTTCGGTACGTATTAGTATGTGAGTGATGTTTAATATATGTCAATGAGTTTATCAAATCATTTGACCATTTCATTGGAAGCTTACACCATAATTTAAAATATTTCTATGTACTCTGCTATAGACATGATTTATTTTTACCACCAATCTCTATAGGCATGACGTCCAAAAAACACTTCATCGTTCCACCCAAACCTTTGCAGAAATCTTTCCAAAATTGCACAATCAACTCTACAAGATCGAAGACCGTCTCCGCAGCATCTACATAATGGAATAGGAAAACGGAAAATAGTAACAGAAAATGAAGTAACATATCAATAAAATCCTATGTTTTT

>SmMEG-26.3

MLLHFLLLFSVFLFHYVDAAETVFDLVELIVQFWKDFCKGLGGTMKCFLDVMPIEIGGKNKSCL

>SmMEG-26.4

ATGAATATGAGTAGTTGTCTGTTGGGCGTAGTATCTATTATTTCAATAtTTTTACTTAGTGGAGTGAATGCTGATAAAGCAATCTTTAATGTCGTAATGTTTATTGTAGAACTATGGAAGGATTTCTGCAAACATTTAAAGGGAACATTCAAATGCTTTTTAGACGAAATTGATCCATCTCTCGGCGGCCAAGGTCATAAATGTACAGAAGTGAATGGAGTGATTTTCAATGTGGAAGAACTTATTTTGGATTTCTGGAAAAATGTATACGAACGCTTAGCTGATACTTGGAAATGCTTATTACAAGTACTATCCAAAGATCTAGGAGGTGAAAATCAAACATGCTACAATTCGACTCAAAGTTAGCGTG

>SmMEG-26.4

MNMSSCLLGVVSIISIFLLSGVNADKAIFNVVMFIVELWKDFCKHLKGTFKCFLDEIDPSLGGQGHKCTEVNGVIFNVEELILDFWKNVYERLADTWKCLLQVLSKDLGGENQTCYNSTQS

>SmMEG26.5

GGTATCAACGCAGAGTACGGGATCAGCTTCAATTGATAATGTTAATTGGTATTGTGTCATTGATTTCAATTTTCTTACTACAAGAGGCAAATGGGGAAGGAGTGATCTTTGATGTGAAAACTCTTATTACGGATACCTGGAAAAATTTATACACACGTTTAGGTGACACTTTCAAATGCTTCTTGAAAAAACTACCTATAAGTCTTGGAGGCGAAAATCGCACATGTATCGAAGAAAAAAGTAAATAAATGTATAATGTCATCGTGATTTGTTGTTTCAAGTGAATTAGTTTTCGATAATTTTATTTCGGTTTATTAGTGTGTTGTTGAAATGTAGATTGATTCAATTTTTGGACACTGTCTTAACACTGTTATGACAGGTGG

>SmMEG-26.5

MLIGIVSLISIFLLQEANGEGVIFDVKTLITDTWKNLYTRLGDTFKCFLKKLPISLGGENRTCIEEKSK

>SmMEG26.6

GCTCTTCCGATCTGGTGAAATAATGCATTCTGATTTTCTACTATCTACGGTAGTGCTAACATTATCACTTATATTACTTGAAGGAATTAATGTAGTGACTGGAGAAGGTGTTATTTTCAACTTCAGGGAACTGTTTACAGATTCTTGGAAGAACTTATATTTACGTTTGAAGGGGACATTCGAATTATTCCTTGATGAAGATTTGGAAAAGAAATATGGAGGAAAATCAAAATAACTTTTAGACTTATGTAATTTCTCTTACTCTGTTTAGTTTTATTTTACTACAGTCCAAAATTGAAATCTGATCTAATCAAAAATTCATGTAGGCAGAGAAATTAATTGAATTGGAAACTTTTGTATATTAAAGTTGCTTTCGGTTGTTTTAATAATGAAGTGCCTCACTACGTGTTTTAAAC

>SmMEG-26.6

MHSDFLLSTVVLTLSLILLEGINVVTGEGVIFNFRELFTDSWKNLYLRLKGTFELFLDEDLEKKYGGKSK

>SmMEG26.7

CGCTATTGGGTAGTTTTATTGTAACATGGATTTTTTTTGTTTATTTAACTTTTATTCAGTTGGATTTTCACGCTATTGGGTAGTTTTATTGTAACATGGATTTTttTTGCCTCCTAGTTCTTCAGGTAGTACTAATAATAAACATCTCCAAGTACCACCTAAGCGTTCATATAAATTGGTCCACAAACCTAAAATAAACTCTACGACATTAAAGATTGCTTTATCAGCATTCACTCCACTAAGTAAAaaTATTGAAATGATGAATACTACGCCCAACAGACAACTACTCATATTCATGATTATTTATTAT

>SmMEG-26.7

MNMSSCLLGVVFIISIFLLSGVNADKAIFNVVEFILGLWTNLYERLGGTWRCLLLVLPEELGGKKNPCYNKTTQ

>SjMEG-26.1

ACGCGGGGATAACCAATGTGTTTATCATGTTACTTGGCTTCAATTTGTTATTCTTAACATTCTTATTACA

TAATGTTAATGGTGAAGGTGTAATCTTTGATGTTGAACTGTTTATTCTAGATCTTTGGACTAATTTTTAT

CAACGTCTAGAGGAAACCATACAATGTTTTTTGGCAGTACTACCCAAACAACTTGGTGGTTCCAGTAAAG

CATGTTATTCAAGCTAACAATAATCCCCATCTAAGGAAATGTCCGTACATTAGAGTTCTACAACTTAAAT

GACAGTCAACATTCCATGGAAGTGTATTTTAATTACTGCTTACTTACATATAAACTATAAACATAGATGT

GAATGTATGTAATGTAATGTAACTTATTGCTTATCAATTACAACTACTTATTATTACGAAAAAAAAAAAA

AAAAAAAAAAAAAAA

>SjMEG-26.1

MLLGFNLLFLTFLLHNVNGEGVIFDVELFILDLWTNFYQRLEETIQCFLAVLPKQLGGSSKACYSS

>SjMEG-26.2

TTAATATATAAAGTTACAAATACACATTTTGATCTGCTTATAGATACCTGTTTTATCCAATAAATAGTGAGTAACAACACTTTAACTTCAAAATCTTTTCAAGGAATTTAACTTTTGGATAATGCATTCTATTTTTCTACAATCTACAATAATTTTCATATTATCGTTAGTATTTCTTCAAGTGATTAAAGGTTCAGGTGACACTGGAGATGCAGTTATTTTCAATTTTAGATTATTCTTTCTAGGAATTTGGGACAATCTGTGTTGTCGTTTACACGGAACTTTCAATTACTTCCTTGATGATTTGCGTAAACGATTCGGGGGATCAAGAGGATGACAATAAACATTTTTTTTCACTTTAACTGGTTATTTTTACTGTGAAAACTTTCATTTTATCATATTTGATCTACTTGAAAAATATATCGAGCAATGGACTTTTGTTACCTTATCGTATTAACTGTAAGCTTCTCCAGTATGTTTATATAACAAAAACCAGTATAGAAACAAGAATAAAAATTAATAAAACAATATTGTTTTGTATTAACAAAAAAAAAAAAAAAAA

>SjMEG-26.2

MHSIFLQSTIIFILSLVFLQVIKGSGDTGDAVIFNFRLFFLGIWDNLCCRLHGTFNYFLDDLRKRFGGSRG

>SjMEG-26.3

CTTCCTTTATCTATTGTCTTCATCTGTATATAACTAGGTGGAGAATTCACTGCATAGTTAAACTTGTCTTAAACTATCGGATTTCATTCATATGTTCTTTAGTATTATTTGGTTACTATCATTATTCTTATTAAAGGAAGTGAATAGTGAAAACACAATATTTGATTTAGTAGAGTTCATTGTACAACTGTGGCTCAATTTCGCCGCTCGTTTAGGAGAAACTTTTAAATGTTATTTAGACATACTTCCTCAACAACTTGGGGGTAAAGACAGAATATGTATAACCAAGAAAAATAGCTCATAACAATTAAGTTCCATAGGAATTCAACTAGATAACCATGAAATAAACAACTTTATTATGAAAAAAATAGTTCATAACAATTAAGTTCCATACGAATTCAACTAGATAACCATGAAATAAACAACTTTATTATGAAAAAAATAGTTCATAACAATTAAGTTCCATAC

>SjMEG-26.3

MFFSIIWLLSLFLLKEVNSENTIFDLVEFIVQLWLNFAARLGETFKCYLDILPQQLGGKDRICITKKNSS

>SjMEG-26.4

AATAGTATGTTGTTTGTTTATTCTGTGCTATTGATTTGCTTGCTTTTATTACATGGAGTGAATGGTGAAGCAATCTTTGATCTTAAGTTGTTTATTCTGGACTTTTGGACCAATTTGGGTAAACGTTTAGGCGAAACTTTGAAATATTTTATAAAAAATTGGAATGGACCTTTTACTCAAGGTGGTGGTCTGATCTTTGATATGGTAACGTTTATTGTCAATATATGGTTAAACTTAAGTGATCGTTTACGAGGGACTTTTGGATGCTTTTTAGATGTACTTGATCCACAACTTGGTGGAAAAAATAAGTCTTGTATATAACGATCTTAAAAACAATAAGTAATAGATTCTCTTGGTGAAAAAAAAAAAAAAAAA

>SjMEG-26.4

MLFVYSVLLICLLLLHGVNGEAIFDLKLFILDFWTNLGKRLGETLKYFIKNWNGPFTQGGGLIFDMVTFIVNIWLNLSDRLRGTFGCFLDVLDPQLGGKNKSCI

>SjMEG-26.5

TGGTGATGGCACAAAATACATAAAGTAATTAACTCATGTGTTTTAGAGGACTGCGTTGTTAGGCGGTTTGTACAATGAGCCTCAGCAACATTCTGTTTAGTTCCTTCCTTGTACTTTCAATATTGTTGGTTCATGGAGTGAATGGTGAAGGCACAATATTCGATCTTGTAACTTTCATTGTAAAACTTTGGGATAATTTTGGTTTACGTTTACGTGATACATTCCAATGTTTTTTGGATGCTTTACCTAAAACACTTGGAGGTAAAAATGAATCATGTAAGGTTTAGTAACAGTCAAATGAAACAGTATATTTTCTGACGATTCTAAAAATGAAATATTTTCATGATTTATTGAATGACTTCTGTATTGAATGTCAAAGGAGTGTTGAATAAATAATAATCAATAAAAAAAAAAAAAAAAAAAA

>SjMEG-26.5

MSLSNILFSSFLVLSILLVHGVNGEGTIFDLVTFIVKLWDNFGLRLRDTFQCFLDALPKTLGGKNESCKV

>SjMEG-26.6

AATTGAAGATAACATCACCATTCGGCTATCTTCAGTATTTCATAGAATTCTCATTAATACACTTATATCTTTGTTGAATATTGAGTGTAACTGATATGTTAATTGTATTTGTGCTACTGTTTTCAGTTGTCCTATTAAAAGAAGTGAATGGTGATGTTATCTTCAATTTGGAAATGTTTATTCTTGACACTTGGACTAATTTGTGTAATCGTTTAGCTGGTACTTTCAGATGTCTTTTGGAGATACTTCCAGTAACACTTGGTGGCAAAGATTGTATATGTATAACTGCAAATAAGACCTCATGTAAAAAAAACAAATAATGTCAATGATCATGTGAATTATTTACAGAAGTTTGAAATAAATTCAATAAAGAATAT

>SjMEG-26.6

MLIVFVLLFSVVLLKEVNGDVIFNLEMFILDTWTNLCNRLAGTFRCLLEILPVTLGGKDCICITANKTSCKKNK

>SjMEG-26.7

CTTTGTTGAATATTGAGTGTAACTGATATGTTAATTGTATTTGTGCTACTGTTTTCAATTGTCCTATTGAAAGAAGTGAATGGTGATGTTATCTTCAATTTGGAGTTGTTTATTCTTGAAATTTGGACTAATTTTTGTGATCGTTTAGGTGGTACTTTCAGATGTTTCTTGGAGATACTTTCAGTAGAACTTGGTGGCAAAGGTTGTACATGTATAGATCGAAATGGGACTTTATGTAAAAATGAAACTAAATCATCATGAAAATGCAGTTGTTTCTTGATTTGATATTCTACATCGATTAAGAACAGCTAATGTCATTGATCATTTTATGTGGATTAGCTATAGAATTTTGAAAGAA

>SjMEG-26.7

MLIVFVLLFSIVLLKEVNGDVIFNLELFILEIWTNFCDRLGGTFRCFLEILSVELGGKGCTCIDRNGTLCKNETKSS

>TrMEG-26.1

CGCTCAAAATCTTGTTTCGATTTCAATGTGGGGATGTTTCATTTACTGAAATAAAAGAGTGACCGCCAATTAAGGTTGGTAAGTACATAATTCGTCAAAAAGAAAGATAAACAACGATCGTGAGACAGACATGCAATGAATACCCCCTACATTACACCAATTCAATGAGAATGCAGTCAGTATTTTTTCTTTTACATTCACACTCAAAACAAATCAAACAAGCATCTCGACGTGATGAGATTTGGCATTTCACTACTCGGATATTTTATTATATTTCTAACAATTCTACTGAATAATGTTAATGGTCAGGATGCAGATCAAGTTATCTTCAATTTGAAAAAGTTTATTCTGGATATATGGTTAGCCCTATGTACCCGACTAGGTGAAACATGGGGCTGCTTATTAGATGCACTGTCAGTGCAACTTGGAGGAAAAGGGAGAACATGTATAAGTAAAAACAATGAGATCATGAAGTAAACATATTTATTCTAGGTTTGTCACCGTGAAGTTAACATCAATCAACCTAAAAAGAATTCAACAAAGCCATTGATTGAGAGAATAATTCGTGAAAAATTCTTAATTGACGATATCACTGAAATATGAAATAAAATGTCCTCCACTCATGAATGCAAAGTATCTCGTATTTCTAGTGCTTTGTATTTGTTAAATAAATATAATACGCATGTTTGAGAAGTCTTTACATATTTCAT

>TrMEG-26.1

MRFGISLLGYFIIFLTILLNNVNGQDADQVIFNLKKFILDIWLALCTRLGETWGCLLDALSVQLGGKGRTCISKNNEIMK

>TrMEG26.2

ACGAAGCATTTCGAAATGATGAGATTAAGTATTTCACAACTTGGATTTCTTATCTTATTTCTCACAATACTATTGAATGAAGTCAATGGTCAAATGGCTGCTCAAGGTCAAATGGGTGTTATCTTCGATTTGAAAATGTTTATTATTGATATCTGGAAAGCGCTTTGCTACCGACTAGTTGATACATGGGGCTGCTTTTTAGATGCGTTACCACGGGAACTTGGAGGAAAACAAAAAACGTGTATAAGTTTAAATCATCAGCAACAATGAATTAGGAATGAGCTCACCATGGATCTCGATTGGGAGAATAATTAATGACAAATAATAGAGACATGATGAAATTGAAATATGAAATACAATTTCTCCGAAACATAAAAACGATTTATTTTGTTGGCTAAAAATAGTACACGTATTTGAAAAACCATTTCTATTTGTAAGCAATAAATAATATCACCTGGAAGTTGAGCAGTTCAGTATGATTAAGGTTTATTTTTCTTTCACAACTAAAACAACATCACTTTGTAGACAAACTTTGCTTAAAT

>TrMEG26.2

MRLSISQLGFLILFLTILLNEVNGQMAAQGQMGVIFDLKMFIIDIWKALCYRLVDTWGCFLDALPRELGGKQKTCISLNHQQQ

>TrMEG26.3

TTCTCTCAAAAAATTGTTTATTTTTCAATTGTTTACTCCGTGGACATAATTAAATTATTTGAAATAGTTTGTCAACAAAATTATCCTGATGGATTTTGGGGTGCTGAATGAAAACATAGAGACTCTTTTCCTCCAAGATTTTTCGGCAACTTATCTAAAAAGCAGCCCCAAGTATCAACTAGTCGGTAACAGGAGGCTTTCCAGATATCCAGAAGGAATTCCTTCAAATTGAAGATCACATCCATGACTGCTTGCTGCTGACCATTGGCTTCATTTAATAGAATTGCGAGAAATATTATACAGAGTCCAAGTATTGAAATACTAAATCCCATCATTTTCAGATGCCTGGTCATCTTCTTG

>TrMEG26.3

MGFSISILGLCIIFLAILLNEANGQQQAVMDVIFNLKEFLLDIWKASCYRLVDTWGCFLDKLPKNLGGKESLCFHSAPQNPSG

>TrMEG26.4

CACACATAAAACGAATAGTTTAGATACACATTTTGAAATATAAATAATTCATATTTCTGTTACCTTACTCTTCTTCTTGTTATTAACATTATTCTTATTTCGAATTGGAACGGGTGTTTGTGATTAAAACAATCTCTAGTCTCATGAAATTTATCAACCAAGAATGTTTATTATTGATTATATTGCTGATAATAACAATCACGATTCATAAAACAAACGGTTCAGCTCAAGATTCACAATCAGGTGTTGTATTCAATCTTCGCCTATATGTTGTACAAGTTTGGTTGAGCTTTTGCAATCATTTCTCTGGAACAGTCAGATACATGTTCGGATGGCTACCTCAAGAACTTGGTGGAAATCCTACGAGTATGAGTGGTTAGGTGTCACATGAAGGCAGCTAGTAATCCTGCAATTCTGACAGAATTTTGAATATAAAATGATTGAAATCTCTACCTTGCTTCCTTTTGCGAATGTCTCAGGTACTGAGTTCTTAGTCTGAAGTCATAATAGACGTAAAAAATAAAGTTTTACTGG

>TrMEG26.4

MKFINQECLLLIILLIITITIHKTNGSAQDSQSGVVFNLRLYVVQVWLSFCNHFSGTVRYMFGWLPQELGGNPTSMSG
