# Supplementary figures and images for: Microexon gene transcriptional profiles and evolution provide insights into blood processing by the Schistosoma japonicum esophagus
Source: PLoS Negl Trop Dis. 2018 Feb 12;12(2):e0006235. doi: 10.1371/journal.pntd.0006235 (PMC5825161; doi:10.1371/journal.pntd.0006235)

## Slide 1
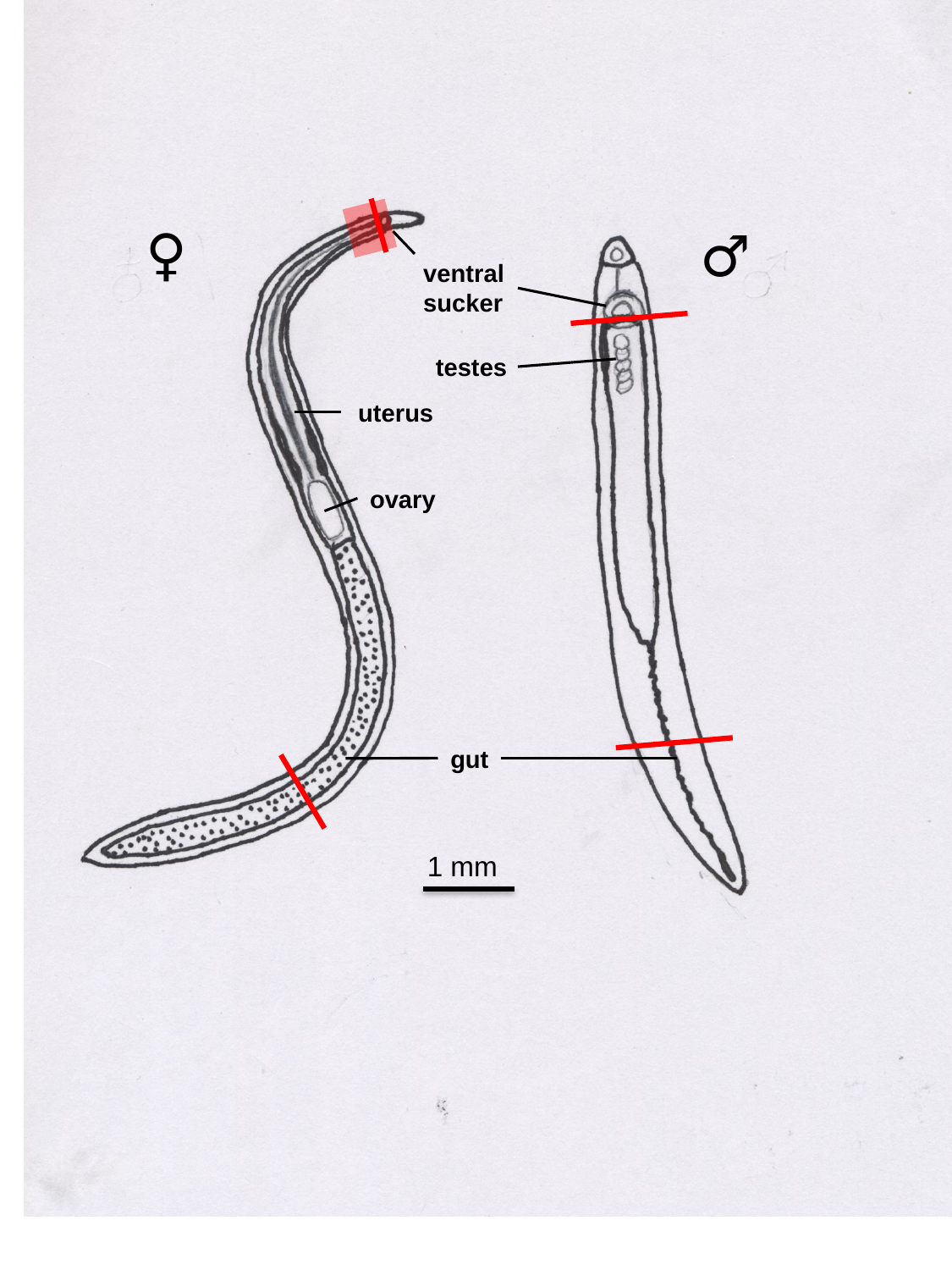

♀
♂
ventral
sucker
testes
uterus
ovary
gut
1 mm

Supplement: S1 Fig — The shaded area in the female indicates the range of the excision point due to the small size of the female head region. (PPTX) [file pntd.0006235.s002.pptx]

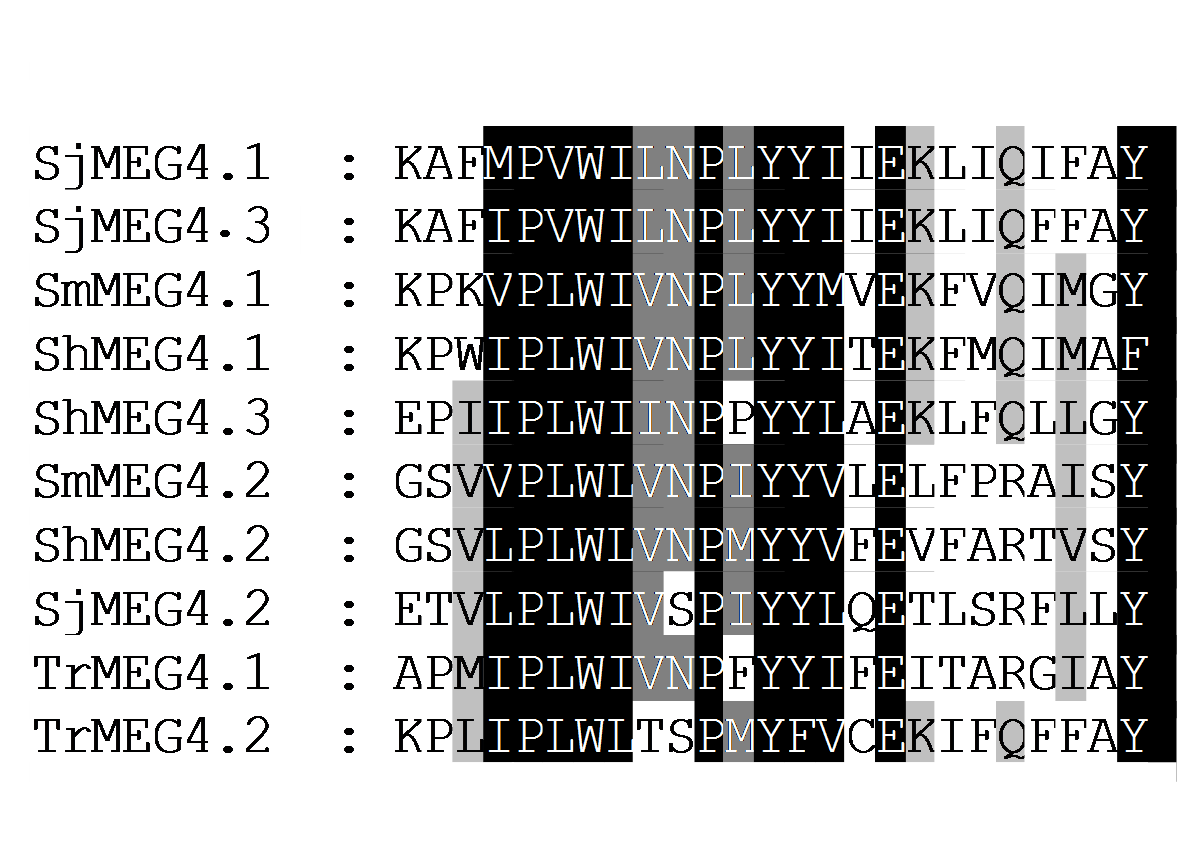

Supplement: S2 Fig — (TIF) [file pntd.0006235.s003.tif]

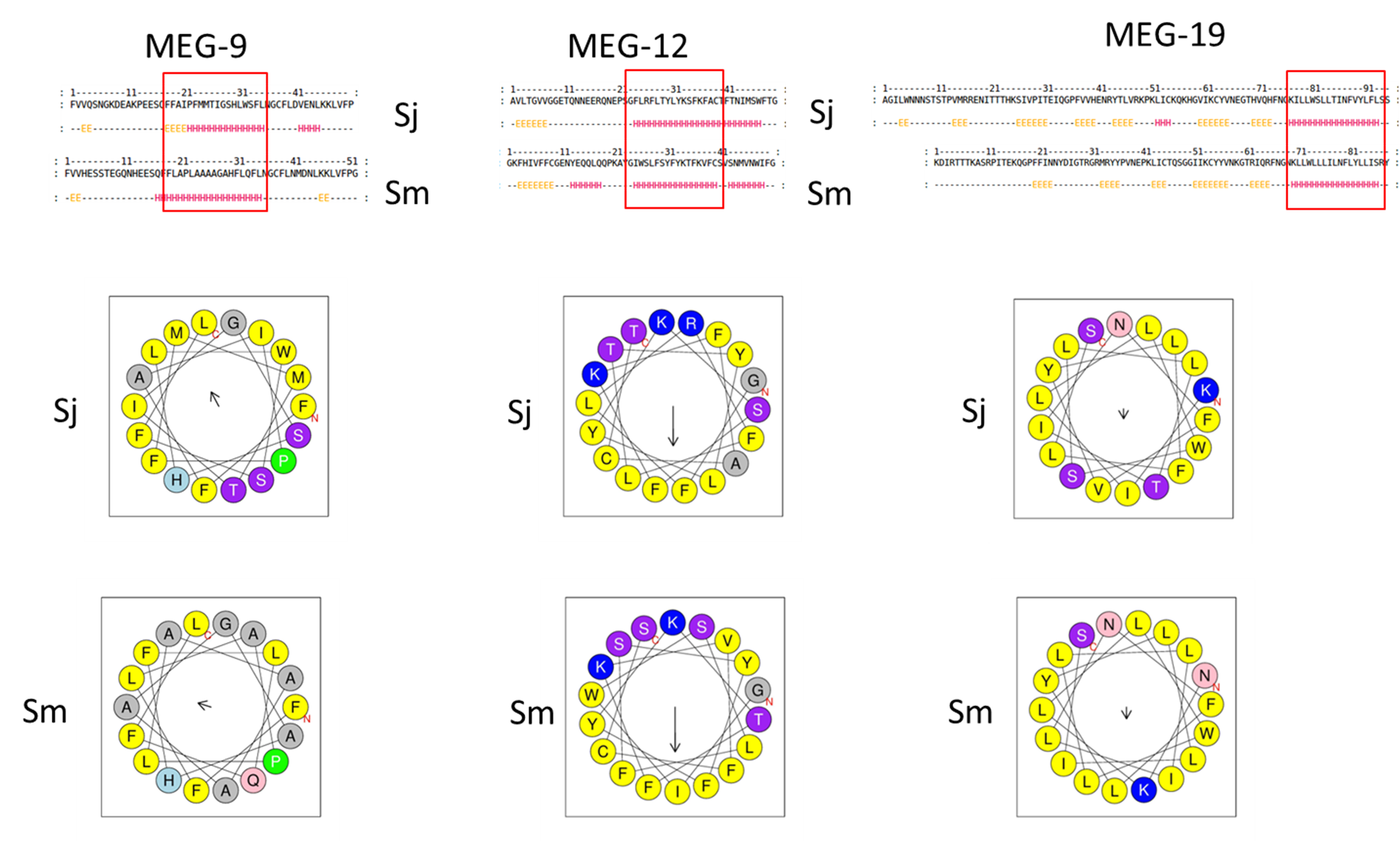

Supplement: S3 Fig — Hydrophobic/amphipathic helix regions within the boxes are denoted HHHH. Helical wheels, drawn using the Heliquest program, display the disposition of residues as follows: apolar = yellow; positively charged = blue; small side chains = grey. The remaining coloured residues represent polar chains. Arrows represent the hydrophobic momentum vector. (TIF) [file pntd.0006235.s004.tif]

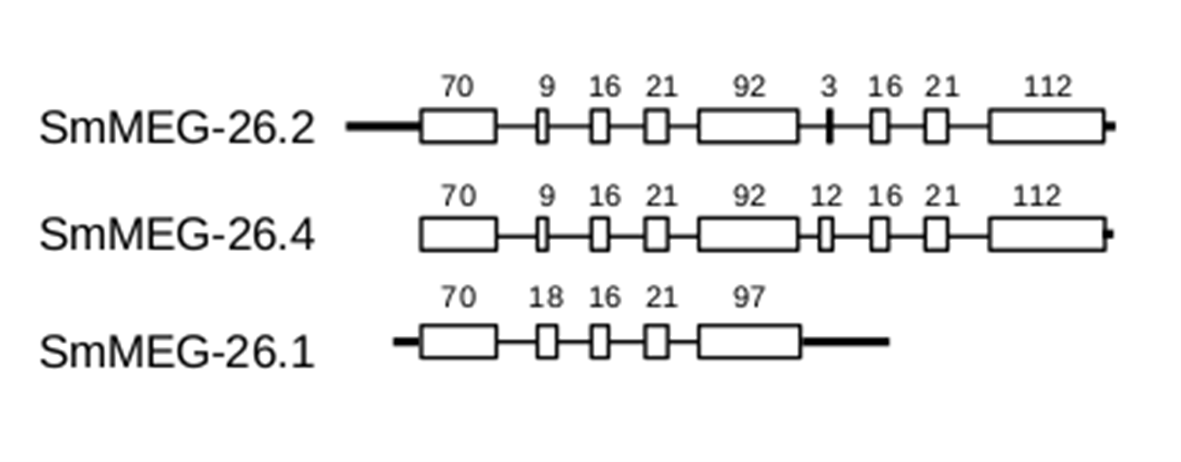

Supplement: S4 Fig — Gene structures of SmMEG-26.2 and 26.4, each with two amphipathic helixes, and MEG-26.1 with a single amphipathic helix are shown. Lengths of white exon boxes are proportional to their nucleotide complement in bp. Thin intron lines are not proportional to their size. Thick lines represent portions of the sequenced UTR and are proportional to their size. Numbers above each exon represent the size of the coding region, including stop codons, expressed in base pairs. (TIF) [file pntd.0006235.s005.tif]

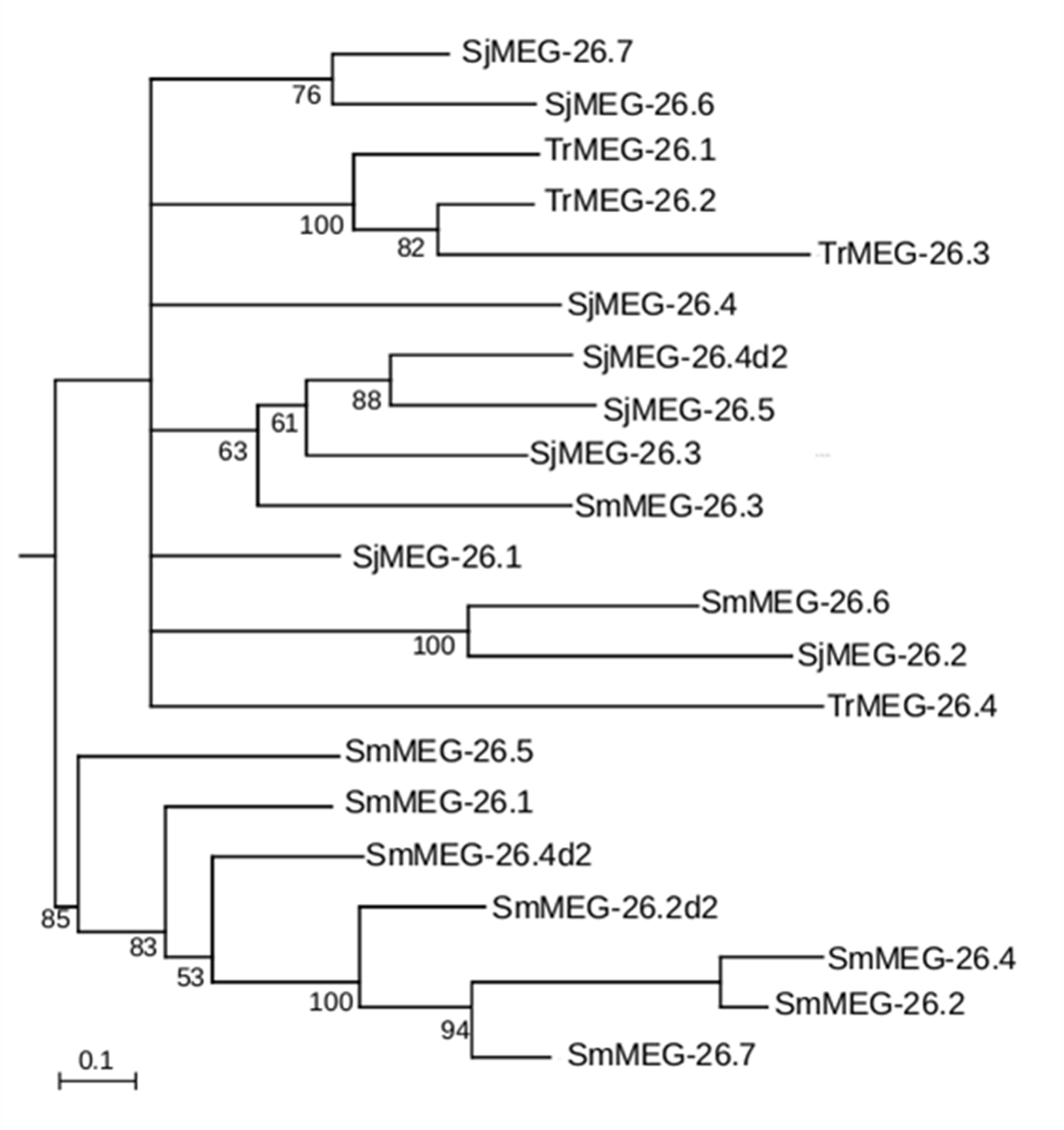

Supplement: S5 Fig — The repeat domains of SmMEG-26.2 and were considered as an independent sequence and given a “d2” suffix. The tree was constructed using Bayesian inference and implemented on MrBayes. Numbers next to each node indicate posterior probabilities. Nodes with posterior probability lower than 50% were collapsed. The tree was rooted at midpoint. (TIF) [file pntd.0006235.s006.tif]

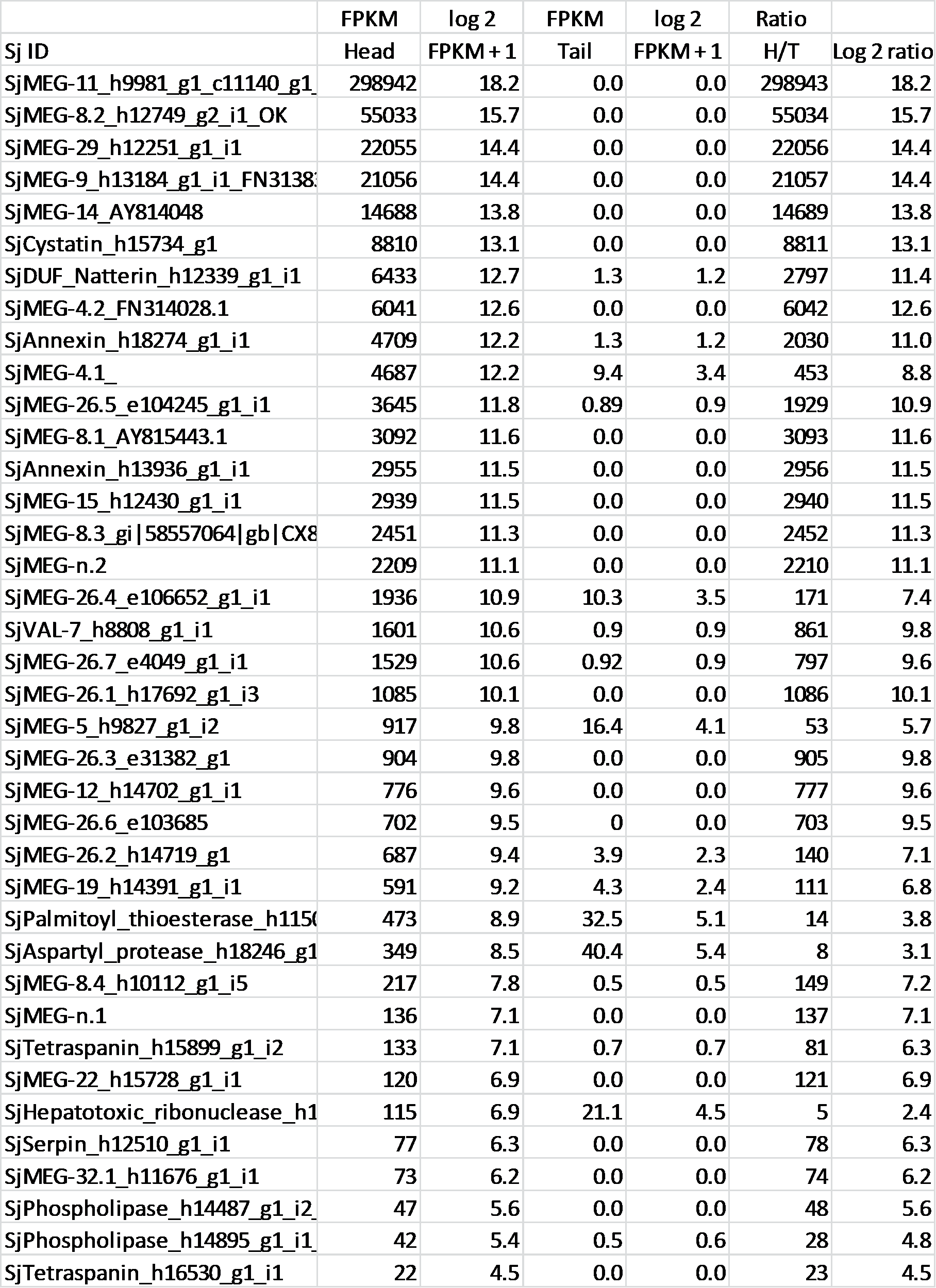

Supplement: S3 Table — (TIF) [file pntd.0006235.s009.tif]
